# Linkage Analysis in Autoimmune Addison’s Disease: NFATC1 as a Potential Novel Susceptibility Locus

**DOI:** 10.1371/journal.pone.0123550

**Published:** 2015-06-04

**Authors:** Anna L. Mitchell, Anette Bøe Wolff, Katie MacArthur, Jolanta U. Weaver, Bijay Vaidya, Sophie Bensing on behalf of The Swedish Addison Registry Study Group, Martina M. Erichsen, Rebecca Darlay, Eystein S. Husebye, Heather J. Cordell, Simon H. S. Pearce

**Affiliations:** 1 Institute of Genetic Medicine, Newcastle University, Newcastle upon Tyne, United Kingdom; 2 Department of Clinical Science, University of Bergen, Bergen, Norway; 3 Institute of Cellular Medicine, Newcastle University, Newcastle upon Tyne, United Kingdom; 4 Royal Devon and Exeter NHS Foundation Trust, Exeter, United Kingdom; 5 Karolinska Institutet, Stockholm, Sweden; 6 Department of Medicine, Haukeland University Hospital, Bergen, Norway; University of Texas MD Anderson Cancer Center, UNITED STATES

## Abstract

**Background:**

Autoimmune Addison’s disease (AAD) is a rare, highly heritable autoimmune endocrinopathy. It is possible that there may be some highly penetrant variants which confer disease susceptibility that have yet to be discovered.

**Methods:**

DNA samples from 23 multiplex AAD pedigrees from the UK and Norway (50 cases, 67 controls) were genotyped on the Affymetrix SNP 6.0 array. Linkage analysis was performed using Merlin. EMMAX was used to carry out a genome-wide association analysis comparing the familial AAD cases to 2706 UK WTCCC controls. To explore some of the linkage findings further, a replication study was performed by genotyping 64 SNPs in two of the four linked regions (chromosomes 7 and 18), on the Sequenom iPlex platform in three European AAD case-control cohorts (1097 cases, 1117 controls). The data were analysed using a meta-analysis approach.

**Results:**

In a parametric analysis, applying a rare dominant model, loci on chromosomes 7, 9 and 18 had LOD scores >2.8. In a non-parametric analysis, a locus corresponding to the HLA region on chromosome 6, known to be associated with AAD, had a LOD score >3.0. In the genome-wide association analysis, a SNP cluster on chromosome 2 and a pair of SNPs on chromosome 6 were associated with AAD (P <5x10^-7^). A meta-analysis of the replication study data demonstrated that three chromosome 18 SNPs were associated with AAD, including a non-synonymous variant in the *NFATC1* gene.

**Conclusion:**

This linkage study has implicated a number of novel chromosomal regions in the pathogenesis of AAD in multiplex AAD families and adds further support to the role of HLA in AAD. The genome-wide association analysis has also identified a region of interest on chromosome 2. A replication study has demonstrated that the *NFATC1* gene is worthy of future investigation, however each of the regions identified require further, systematic analysis.

## Introduction

Autoimmune Addison’s disease (AAD) is, a rare endocrine condition with a prevalence in Caucasian European adults of up to 140 per million [1]. It is highly heritable compared to other autoimmune conditions, with an estimated sibling recurrence risk ratio (λs) of between 160–210 [2]. Its rarity and strong genetic aetiology make it possible that there may be one or two highly penetrant variants conferring disease susceptibility in humans. Alternatively, AAD may arise as a result of a number of rare but high risk variants within a single, as yet unknown, gene. However, we know relatively little about its genetic architecture and its rarity confounds attempts at investigation because large AAD cohorts suitable for a powerful genetic study are scarce. Genetic variants known to contribute to AAD susceptibility include those at the *MHC* [3], *MICA* [4,5], *CIITA* [6,7], *CTLA4* [8,9], *PTPN22* [10,11,12], *CYP27B1* [13], *NLRP-1* [14], *STAT4* [15], *GATA3* [15] and *CD274* [16] loci. These have been detected through candidate gene association studies conducted mostly on relatively small patient cohorts. However, by their nature, candidate gene studies select a limited and skewed series of genes, shaped by the investigators’ preconceptions, and are therefore unlikely to elicit novel pathogenic mechanisms of disease. If a large cohort of unrelated AAD patients and healthy controls could be collected together, a genome-wide association study could be conducted to shed an unbiased light on the underlying genetic aetiology of AAD. This approach has been used for other complex autoimmune conditions, for example type 1 diabetes [17]. However, to date, even collaborative efforts to collect enough AAD samples together for this have not resulted in a large enough cohort for an initial genome-wide study and a replication study. Linkage studies are an alternative, powerful, and hypothesis-free means of identifying previously unsuspected genetic susceptibility loci. In this study, we have used linkage analysis in well characterised multiplex AAD pedigrees, that is, families comprising two or more individuals with AAD, for the first time to look for novel genetic susceptibility loci. We have also used association analysis to compare the AAD cases from the pedigrees to 2706 controls from the UK Wellcome Trust Case Control Consortium (WTCCC) 1958 Birth Cohort [18]. Finally, we have attempted to replicate some of the linkage findings in unrelated AAD case-control cohorts.

## Ethical Approval and Study Subjects

Ethical approval for this work was obtained in each participating country as follows: Stockholm, Sweden—Regionala etikprovningsnamnden i Stockholm Dnr 2008/296-31/2; Bergen, Norway—Regional Ethics Committee West; Newcastle, UK—Leeds (East) Research Ethics Committee, 2005 (REC reference number 05/Q1206/144). Informed, written consent was sought from each study participant at all centres with the exception of the Norwegian controls. These samples were gathered through the national blood donor scheme. All blood donors are informed of ongoing research through written information and are given the opportunity to opt out should they wish to do so. All Norwegian control samples collected in this way are anonymised at source.

23 Caucasian multiplex AAD families from the UK and Norway, comprising 117 individuals, were included in the final analysis. None had autoimmune polyendocrinopathy syndrome type 1 features (hypoparathyroidism, candidiasis). Details of these families are included in Table 1. In the 23 families as a whole, 7 individuals with AAD were 21-hydroxylase (21OH) autoantibody negative, and 12 could not be tested due to lack of serum. These individuals all had either a personal history of autoimmune thyroid disease or type 1 diabetes, a history of these conditions in a first degree relative or a close relative with positive 21OH autoantibodies, with the exception of two sisters from one UK family. These factors combine to make a diagnosis of AAD likely, even in those without confirmed 21OH autoantibodies. The two siblings who did not meet the above criteria, did however both have ankylosing spondylitis in addition to AAD, a disease with a probable underlying autoimmune aetiology.

**Table 1:** Demographics of the 23 multiplex AAD kindreds from the UK and Norway.

	UK multiplex AAD families	Norwegian multiplex AAD families
Number of families	12	11
Total number of individuals	56	61
Number of cases (number of females)	25 (12)	25 (17)
Mean age of onset of AAD (years); age range (years)	39: 18–67	30: 10–67
Number of controls (number of females)	31 (17)	36 (17)
Number of affected sibling pairs	8	4
Number of affected parent child pairs	3	1
Number of affected sibling trios	1	1
Number of affected other relationships eg avuncular, cousins, sib-pairs in multiple generations etc	0	5
Number of AAD cases with autoimmune comorbidities	14^&^	10^£^
Number of 21OH autoantibody positive/negative/untested AAD cases	9/6/10	22/1/2
Number of controls with autoimmune comorbidities	8^*^	4^$^

Autoimmune comorbidities in the UK AAD cases (^&^) included autoimmune thyroid disease (n = 7), type 1 diabetes (n = 6), seronegative (n = 3) arthritis, pernicious anaemia (n = 2), vitiligo (n = 1), rheumatoid arthritis (n = 1), Sjögren’s (n = 1), SLE (n = 1), male hypogonadism (n = 1), premature ovarian failure (n = 1). Autoimmune comorbidities in the UK controls (^*^) included autoimmune thyroid disease (n = 4), type 1 diabetes (n = 1), pernicious anaemia (n = 1), vitiligo (n = 1) and coeliac disease (n = 1). Autoimmune comorbidities in the Norwegian AAD cases (^£^) included autoimmune thyroid disease (n = 9), type 1 diabetes (n = 4), vitiligo (n = 2), rheumatoid arthritis (n = 2), coeliac disease (n = 2), pernicious anaemia (n = 1), gonadal failure (n = 1), Sjögren’s syndrome (n = 1). Autoimmune comorbidities in the Norwegian controls (^$^) included autoimmune thyroid disease (n = 4) and diabetes (type not specified, n = 1).

For the genome-wide association study, genotype data from 2706 controls (1310 females) from the WTCCC publicly available UK 1958 Birth Cohort was used.

For the replication study, DNA was available from unrelated AAD case-control cohorts from the UK (346 AAD, 367 controls), Norway (384 cases, 384 controls) and Sweden (up to 367 AAD, 366 controls).

All single nucleotide polymorphism (SNP) locations refer to HapMap data release number 28, on NCBI B36 assembly [11].

## Methods

Genotyping for the linkage study and genome-wide association study was undertaken on the Affymetrix SNP 6.0 array, according to manufacturer’s instructions, in two batches. The first batch was genotyped at Almac Diagnostics (Craigavon, Northern Ireland—UK families 1 to 9 inclusive) and the second at AROS Applied Biotechnology (Aarhus, Denmark—Norwegian families 10 to 20 and UK families 21 to 23 inclusive). To ensure genotyping fidelity, one sample was genotyped twice, once in each batch and the results were found to be comparable. Called genotype results were returned for each genotyped individual by the provider. Following the linkage and association analyses, results were analysed. A replication study was then planned to further investigate two of the linked regions. For the replication study, 64 HapMap tag SNPs were selected and genotyped at CIGMR, Manchester, on the Sequenom iPlex platform as per the manufacturer’s instructions in UK, Norwegian and Swedish AAD case-control cohorts (primer sequences available in S1 Table).

### Data cleaning and quality control

Quality control (QC) measures taken for each aspect of the study are shown in S2 Table.

### Data analysis

Following data QC, 14,771 informative SNPs (approximately 4 SNPs per centimorgan (cM)) were selected for linkage analysis in Merlin [19]. The information content of the SNP marker map was checked, using Merlin, to ensure even marker coverage across the autosomes. A parametric analysis, applying a rare dominant model (assuming a disease allele frequency in the population of 1 in 10,000 (0.0001) and assuming a disease penetrance of 0.001 (0.1%) if 0 risk alleles are present and 0.999 (99.9%) if 1 or 2 risk alleles are present) and a non-parametric analysis were performed on the autosomes. The analysis was also repeated using a denser SNP marker map of 36,775 SNPs and a sparser SNP marker map of 7429 SNPs (approximately 2 SNPs per cM): the results were comparable. By convention, parametric linkage results are reported in LOD (logarithm of odds) scores. HLOD (heterogeneity LOD) scores, which allow for locus heterogeneity in a linkage analysis, are also reported. When the LOD and HLOD scores are the same, all families are contributing to the result. In the case of the LOD and HLOD scores differing, an estimate of the proportion of families contributing to linkage is given as α. Non-parametric linkage results are reported in terms of the model applied, either the Kong and Cox linear model, or the later improved exponential model [20]. In this study, all linkage signals generating a LOD or HLOD greater than 2.0 are reported. A LOD score of >2.5 was taken as being highly suggestive of linkage while a LOD score of >3.0 was taken as convincing evidence of linkage. We note that the “highly suggestive” threshold used here is slightly more stringent than the “suggestive” threshold used by Lander and Kruglyak [21], while the “convincing” threshold used here is slightly less stringent than the “significant” threshold that they proposed.

To compare genetic variation in the AAD families with that seen in a population-based control sample, a dense, quality controlled marker map (595,118 SNPs) was used for a genome-wide association analysis using EMMAX software [22]. EMMAX (Efficient mixed-model association expedited) is freely available software that is based upon EMMA (efficient mixed-model association), a variance component approach, which allows for sample structure by explicitly accounting for pairwise relatedness between individuals. The 50 affected family members were compared as cases to control genotype data from 2,706 controls from the UK 1958 Birth Cohort. A genome-wide significance level of P <5x10^-7^ was chosen to account for multiple testing.

Genotype and allele data generated from the replication study for each of the 3 national cohorts were used to construct 3 x 2 and 2 x 2 contingency tables and then analysed using chi squared tests. To allow the national cohort data to be analysed as a whole, in order to increase the study power, results were combined using meta-analysis and the Revman 5 program [23] with a random effects model to account for heterogeneity.

### Power estimations

The SLINK program [24] was used to estimate power for the linkage analysis. Power calculations were based on the most powerful analysis to be undertaken. In a parametric analysis, assuming a rare dominant model and that 75% of families are linked, the study has 77% power to detect a locus with an HLOD score of 3.0, and 98% power to detect a locus with an HLOD score of 2.0. For the genome-wide association analysis, power calculations were performed using QUANTO [25]. Assuming a minor allele frequency (MAF) of 0.3 and applying a genome wide α of 5x10^-7^, the study has 52% and 92% power to detect loci with odds ratios of 3.0 and 4.0 respectively. Assuming a lower MAF reduced study power. QUANTO [25] was also used to estimate the power of the replication study. Assuming a MAF of 0.3 and α 0.05, the replication study has 57% power to detect a locus with an odds ratio of 1.2, 98% power to detect a locus with an odds ratio of 1.4 and 100% power to detect a locus with an odds ratio of 1.6 or greater. Assuming a lower MAF reduced study power.

## Results

### Linkage analysis results

**Fig 1:**
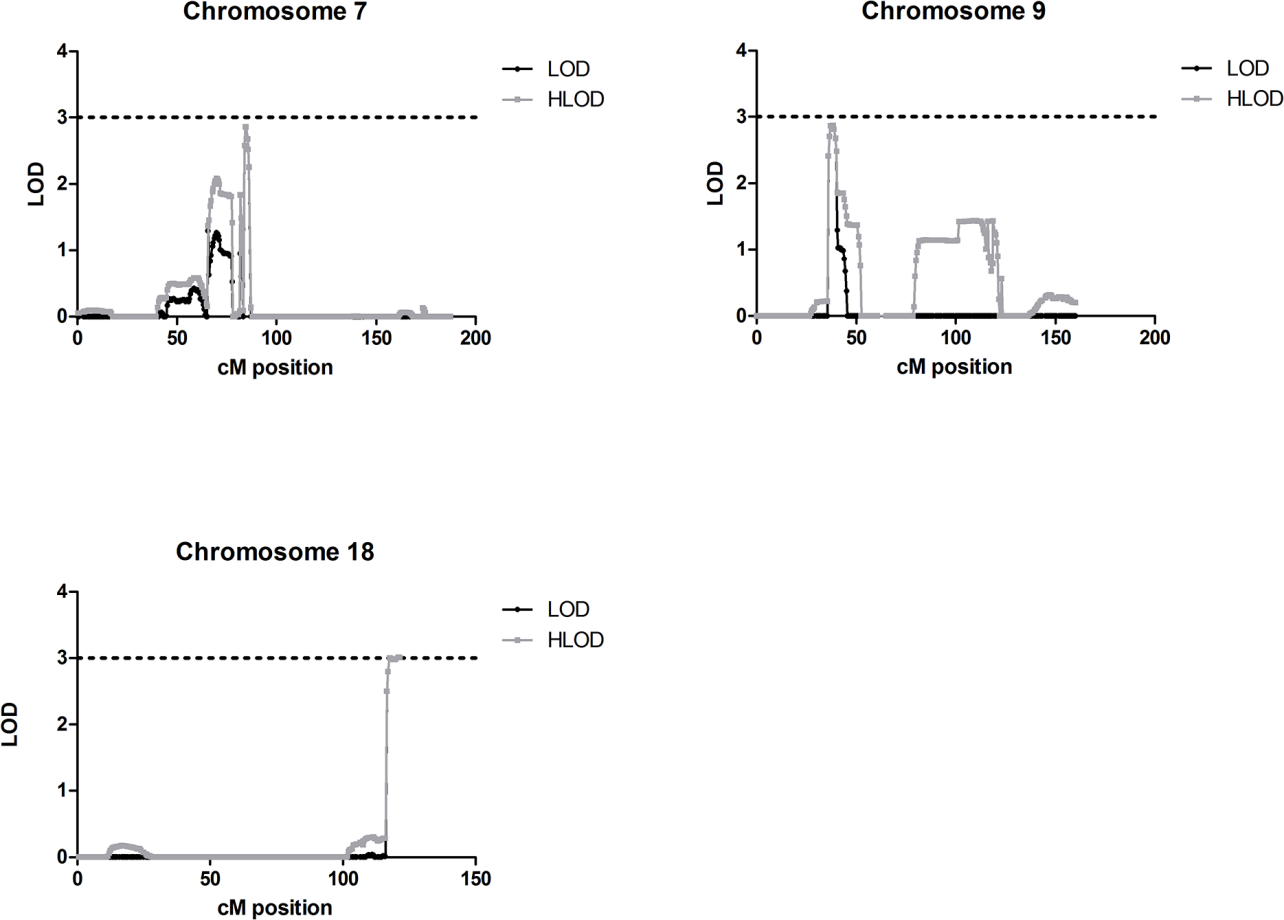
Graphical representation of parametric linkage results suggestive of linkage (LOD score >2.5) in the multiplex AAD families, assuming a rare dominant model. Graphical representation of parametric linkage results in the multiplex AAD families, assuming a rare dominant model. The LOD/HLOD score is on the y axis and the cM position is on the x axis. The dotted black line shows the LOD/HLOD threshold of 3.0, taken as convincing evidence of linkage. LOD scores are shown with black lines and HLOD scores with grey lines. Linkage peaks of LOD/HLOD greater than 2.5 were observed on chromosomes 7 (upper left panel), 9 (upper right panel) and 18 (lower left panel).

Applying a rare dominant model, three loci on chromosomes 7, 9 and 18 had LOD/HLOD scores of greater than 2.5 (Fig 1). The maximum LOD score was observed within a linkage peak on chromosome 18, between 116.5 and 121.9 cM (corresponding to 75241668–77950543 base pairs (bp)). Within this peak, a maximum LOD and HLOD score of 3.00 was seen at marker *SNP_A-8291421* (119.6 cM, *rs1113678*, 76554812 bp). On chromosome 9, a linkage peak was observed between 36.0 and 40.4 cM (17486802–19751149 bp), with a maximum LOD and HLOD score within this peak of 2.90 at marker *SNP_A-1996138* (38.6 cM, *rs10123624*, 19025385 bp). On chromosome 7, a maximum LOD and HLOD score of 2.88 was seen at marker *SNP_A-4232044* (82.6 cM, *rs10263367*) at position 70082089 bp within a linkage peak spanning 82.4–86.2 cM (70020160–73809454 bp). A second, smaller linkage peak between 69.8 and 71.7 cM (47565504–49876993 bp) was also seen on chromosome 7. Within this peak, a maximum HLOD of 2.09 at marker *SNP_A-2279338* (71.0 cM, *rs13228770*, 49457067 bp) was observed (estimated proportion of linked families (α) 0.66). At this locus, the maximum LOD score was 1.28. Applying this model, no other locus had a LOD/HLOD score of greater than 2.0.

**Fig 2:**
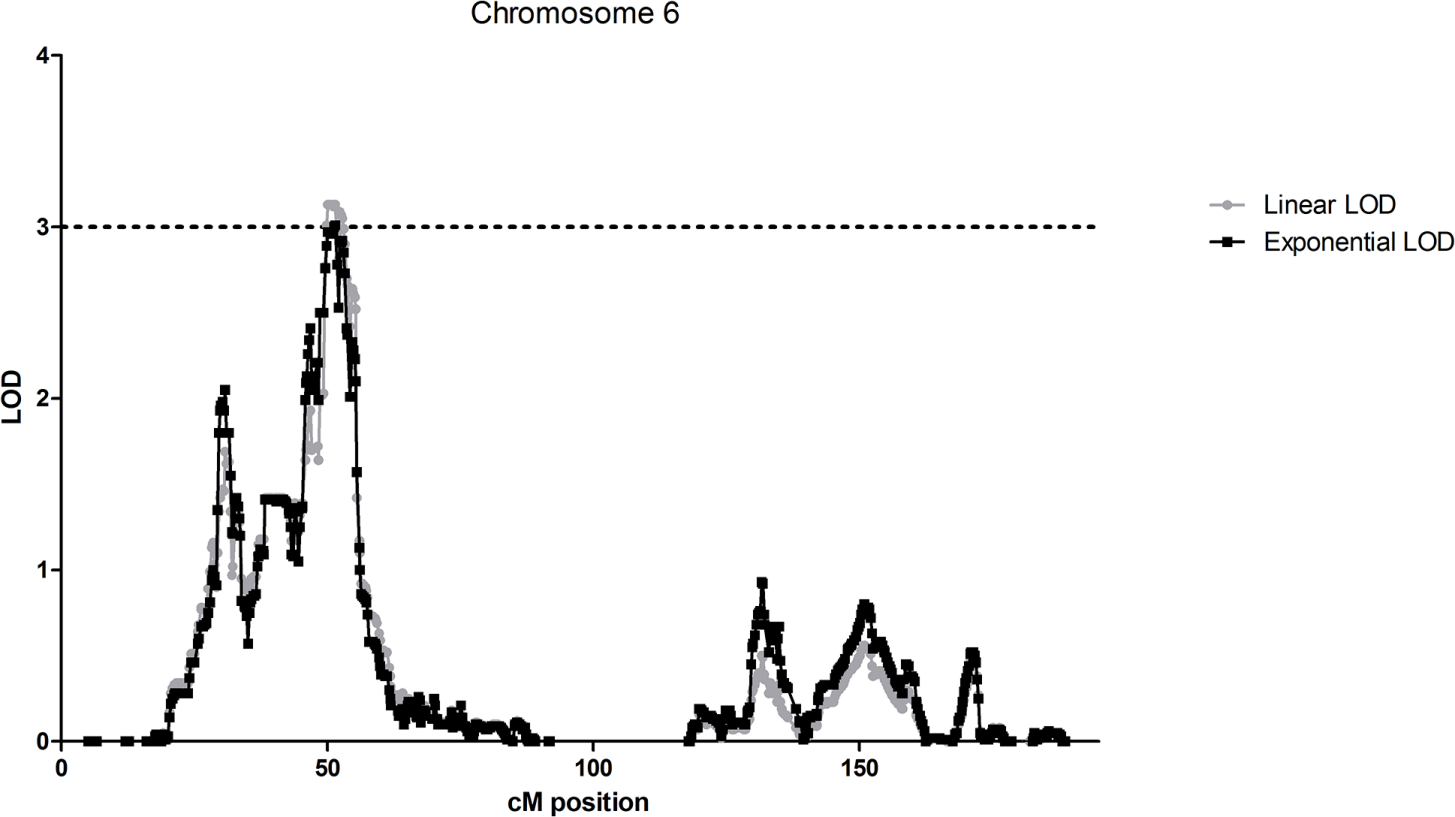
Graphical representation of non-parametric linkage results convincing of linkage in the multiplex AAD families. Graphical representation of non-parametric linkage results in the multiplex AAD families. The LOD score is on the y axis and the cM position is on the x axis. The dotted black line shows the LOD threshold of 3.0, taken as convincing evidence of linkage. Exponential LOD scores are shown with black lines and linear LOD scores with grey lines. A linkage peak of LOD greater than 3.0 was observed on chromosome 6 only.

In a non-parametric analysis, which effectively excludes the parent-offspring pairs (18 kindreds informative), one locus on chromosome 6 had a LOD score of greater than 3.0 (Fig 2). Here, a large linkage peak was seen from 46.0 to 55.4 cM (22375648–35968100 bp). The maximum LOD score, applying the Kong and Cox exponential model, was 3.01 at 51.5 cM (*SNP_A-1923640*, *rs2072633* at 31919578 bp). At this locus, the linear LOD score was 3.13. In this analysis, no other loci had LOD scores greater than 2.0. A full list of genes in the linked regions can be found in S3 Table.

### Genome-wide association analysis results

In the genome-wide association analysis, 17 SNPs were associated at a level of genome-wide significance (P<5 x 10^–7^) (Table 2). The majority of these were individual SNPs likely representing spurious association due to genotyping error, as SNPs in tight linkage disequilibrium (LD) with them (r^2^ >0.8) were not similarly associated (data not shown). However, association was noted with a cluster of five intergenic SNPs in tight LD (r^2^ >0.8), spanning 228 kilobases (kb) on chromosome 2, with maximal association at *rs10495950* (P 9.7 x 10^–8^). In addition to these 5 SNPs reaching genome-wide significance, a further 5 SNPs in LD with these (r^2^ >0.6) were associated with AAD, although to a lesser degree, not reaching genome-wide significance. In addition, a pair of SNPs on chromosome 6 (*rs2187668*, P = 3.0 x 10^–7^; *rs1150753*, P = 4.4 x 10^–7^) were also associated with AAD. Again, an additional 7 SNPs in LD with these (r^2^ >0.6) were also associated, but the degree of association did not reach genome-wide levels of significance.

**Table 2:** Association analysis results comparing the 50 AAD family cases to 2706 1958 UK birth cohort controls.

Chr	rs ID	Position (bp)	P value	Position
2	*rs11681243*	48104650	1.7 x 10^–7^	Intergenic
2	*rs7561696*	48157345	1.8 x 10^–7^	Intergenic
2	*rs13431982*	48286415	3.3 x 10^–7^	Intergenic
2	*rs10495950*	48288258	9.7 x 10^–8^	Intergenic
2	*rs17325209*	48332704	2.1 x 10^–7^	Intergenic
2	*rs1406242*	151151406	5.9 x 10^–20^	Intergenic
3	*rs7632505*	124220987	4.3 x 10^–9^	SEMA5B (semaphorin 5B)
3	*rs16840699*	134920281	2.6 x 10^–10^	Intergenic
6	*rs1150753*	32167835	4.4 x 10^–7^	TNXB (Tenascin B isoform 1)
6	*rs2187668*	32713852	3.0 x 10^–7^	HLA-DQA1
6	*rs15680*	58380385	1.5 x 10^–12^	Glucuronidase beta-like 2)
8	*rs1426192*	21392622	3.4 x 10^–10^	Intergenic
13	*rs873294*	109965408	5.4 x 10^–24^	Intergenic
14	*rs8007744*	27399226	1.1 x 10^–18^	Intergenic
18	*rs7229302*	11498894	9.1 x 10^–16^	Intergenic
21	*rs3787764*	37154098	6.2 x 10^–25^	HLCS (Holocarboxylase synthetase)

All SNPs with a P value less than 5 x 10^–7^ are shown in chromosome order. The final column shows the SNPs in relation to genes and non-coding RNAs. Intergenic SNPs are those not in genes or non-coding RNAs.

### Replication study rationale and results

As the linkage peaks and the associated regions were large and numerous, an initial replication study to look at just two regions of interest was undertaken, with plans to look at the other regions of interest in the future. Chromosome 6, which contained both linked and associated loci, was eliminated from further analysis because of the proximity of the findings to the MHC. MHC is already known to be strongly associated with AAD and most other autoimmune conditions and more novel findings were sought. The linkage peak on chromosome 18 was selected as the first area for more detailed analysis, as this had the highest LOD score and the region of linkage was close to a plausible candidate gene, nuclear factor of activated T-cells, cytoplasmic, calcineurin-dependent 1 (*NFATC1*). The linkage peak on chromosome 7 was then also chosen for further investigation over the linkage peak on chromosome 9, as the region of interest is very gene-rich.

For the replication study, 64 HapMap tag SNPs in the linked regions on chromosomes 7 (70020160–73809454 bp) and 18 (75241668–77639585 bp) were selected and genotyped on the Sequenom iPlex platform in UK, Norwegian and Swedish AAD case-control cohorts (primer sequences available on request).

The genotype frequencies from the national cohorts and meta-analysis results are shown in S4 Table. In the meta-analysis, three independent SNPs (r^2^ <0.2), all on chromosome 18, were associated with AAD (P<0.05). Maximal association was seen at *rs7236339* where the minor A allele was present in 20.4% of the cases across the three national cohorts compared to 24.1% of the controls (438/2150 cases, 528/2190 controls: P = 0.004). Nominal association was also noted at *rs754093* where the minor G allele was present in 47.1% of cases compared to 43.7% of controls (1034/2194 cases, 977/2234 controls: P = 0.02) and at *rs8091998* where the minor T allele was present in 20.2% of cases compared to 23.0% of controls (435/2150 cases, 503/2190 controls: P = 0.04). Table 3 contains a summary of significant results from the three analyses contained within this study.

**Table 3:** Summary of study results.

Study	Analysis	Chromosomal segment linked/associated	Most linked/associated marker (position)	Maximum LOD/HLOD or P value
Linkage study	Non-parametric	Chr 6: 22.4–36.0 Mb	*rs2072633* (32.0 Mb)	Linear LOD 3.13/exponential LOD 3.01
	Parametric, rare dominant	Chr 18: 75.2–78.0 Mb	*rs1113678* (76.6 Mb)	LOD/HLOD 3.00
		Chr 9: 17.5–19.8 Mb	*rs10123624* (19.0 Mb)	LOD/HLOD 2.90
		Chr 7: 70.0–73.8 Mb	*rs10263367* (70.1 Mb)	LOD/HLOD 2.88
Association study	50 AAD, 2706 WTCCC controls	Chr 2: 48.1–48.3 Mb	*rs10495950* (48.3 Mb)	P = 9.7 x 10^–8^
		Chr 6: 32.2–32.7 Mb	*rs2187668* (32.7 Mb)	P = 3.0 x 10^–7^
Replication study meta-analysis	Maximum 1097 AAD, 1117 controls	Chr 18: 74.5–76.1 Mb	*rs7236339* (75.7 Mb)	P = 0.004
		Chr 18: 74.5–76.1 Mb	*rs7231100* (74.5 Mb)	P = 0.004
		Chr 7: 69.4–70.9 Mb	*rs12698902* (69.5 Mb)	P = 0.01

For the linkage study, only loci with a maximum LOD/HLOD of greater than or equal to 2.50, suggestive of linkage, are shown. For the association study, only loci where at least two SNPs in LD (r2 >0.8) reached genome-wide significance (P <5 x 10^–7^) are shown. For the replication study meta-analysis, all statistically significant results (P <0.05) are presented.

## Discussion

This is the first linkage study in AAD and takes advantage of a unique resource of carefully phenotyped multiplex AAD families from both the UK and Norway. Importantly, in a non-parametric analysis, a linkage peak with a linear LOD score of 3.13 was seen on chromosome 6, corresponding to the HLA region, a known susceptibility locus for AAD. This finding demonstrates that the non-parametric analysis within the linkage study was sufficiently powerful to detect a true AAD locus with an odds ratio previously estimated to be between 3 and 15 [26,27]. Applying a rare, dominant model, linkage analysis implicates regions of chromosomes 7, 9 and 18 in susceptibility to AAD.

Assessing the pedigrees included in this analysis, no unifying mode of disease inheritance was apparent across all families. In any linkage analysis, this apparent heterogeneity can be allowed for either by using a non-parametric approach, or by using a parametric approach with a “best fit” model and assessing the proportion of linked families, represented by α. The non-parametric approach, which assesses sharing of alleles that are identical by descent (IBD) and therefore by necessity must exclude affected parent-offspring pairs, loses some power as a result of the reduced number of kindreds suitable for the analysis. In contrast, the parametric approach allows all pedigrees to be included, thus increasing study power, and the calculation of the HLOD allows for genetic heterogeneity between the kindreds. At the linkage peaks on chromosomes 7, 9 and 18, all families were contributing to the results and therefore the observed LOD and HLOD scores were the same.

Aside from linkage to, and association with, the MHC region of chromosome 6, the linkage peaks and clusters of associated SNPs detected in the linkage and genome-wide studies presented here do not correspond to known, widely replicated AAD susceptibility loci such as *CTLA4* and *PTPN22*. At loci where the previously reported associations are modest, this may be because parts of this study, in particular the genome-wide association study, are underpowered. However, it is perhaps not surprising given that the hypotheses underlying linkage and association analyses are different. Linkage analysis could be positive if distinct rare variants in a single gene were conferring disease susceptibility in several families, however these variants would not be replicated by association analysis due to their rarity, as association analysis relies on the common disease, common variant (CDCV) hypothesis [28]. It is also possible that the genetic architecture of familial and sporadic forms of AAD differ, meaning that case-control association analysis results may not be replicated in a linkage analysis and *vice versa*. However, the finding of linkage with the *HLA* region suggests that the multiplex AAD families do at least share some susceptibility loci with sporadic cases.

A genome-wide association analysis, comparing the 50 individuals with AAD from the pedigrees to 2706 UK 1958 Birth Cohort controls, revealed two clusters of associated SNPs on chromosomes 6 and 2 meeting genome-wide levels of significance. The associated SNPs on chromosome 6 correspond to the *HLA* region (*rs1150753* is located in the Tenascin B gene while *rs2187668* is found in the *HLA-DQA1* gene) and again validates the approach. In addition, a cluster of five SNPs spanning 230 kb on chromosome 2 were also associated. These SNPs are located between the *FBXO11 (*F-box only protein 11) and *FOXN2* (Forkhead box N2) genes, both of which are expressed in adrenal cortex. The *FBXO11* protein product contains arginine methylation domains [29] and regulates the TGFβ signalling pathway [30]. The *FOXN2* gene encodes a forkhead domain DNA binding protein. However, this is a region of extended LD, which includes other genes expressed in adrenal cortex including mutS homolog 6 *(MSH6)*, a DNA repair gene [31] protein phosphatase 1, regulatory subunit 21 *(PPP1R21)*, a gene involved in the regulation of cell signalling and DNA repair [32] and stonin 1 *(STON1)*, involved in molecule trafficking [33]. Based on function alone, the *FBXO11* gene is the most likely candidate in AAD; however the true association could lie with any of these genes, or with a variant in a non-coding regulatory element. This region therefore requires further investigation.

One significant limitation of the genome-wide association study is that a publicly available UK cohort of controls was used as a comparator, however just over half of the cases from the multiplex families were from Norway. Using the unaffected multiplex family relatives as controls would have been one way of controlling for this bias, however the small size of the cohort meant that this analysis would be greatly underpowered. The UK WTCCC controls were selected for this study as the data are freely available, the demographics of the control population are well described and the data have undergone strict quality control measures and are therefore known to be robust. A similar Norwegian control cohort that could be used for such a comparison is not freely available. Furthermore, if the 50 familial AAD cases were to be divided into individual UK and Norwegian cohorts for separate analyses, the study would lose significant power. Moreover, we have recently demonstrated that the UK and Norwegian populations are genetically similar [15] and therefore using a large UK control cohort is not unreasonable in this case, but should be considered in interpreting the results of the analysis.

A replication study, looking at 64 SNPs in the linkage peaks of chromosomes 7 and 18, was conducted in AAD case-control cohorts from the UK, Norway and Sweden (although we note that, strictly speaking, an association result cannot be considered a direct replication of a linkage result). To gain maximum study power, a meta-analysis was performed across the 3 national cohorts, using a random effects model to allow for any heterogeneity. Nominal association was observed with three independent SNPs on chromosome 18 and AAD. One of these, *rs754093*, encodes a non-synonymous variant (*pCys751Gly*) in exon 9 of the *NFATC1* (nuclear factor of activated T-cells, cytoplasmic, calcineurin-dependent 1) gene. This amino acid residue is conserved across many species including primates and rodents. SIFT analysis, which uses an algorithm to predict whether an amino acid change will disrupt a protein, predicts that this is a deleterious mutation [34]. *NFATC1* is a plausible candidate gene for AAD as it is expressed in adrenal cortex and encodes a transcription factor which plays a central role in gene transcription during the immune response [35]. The two other nominally associated markers are intergenic.

Unfortunately, linkage analysis has low resolution and the linkage peaks generated in the study of the multiplex families were large and numerous. We selected two linkage peaks for further study and selected these on the basis that the linked regions were gene-rich and contained some plausible candidates for AAD which had not previously been investigated. In the replication study, despite focussing our efforts on only two of the four linkage peaks, we were still unable to account for all variation in the regions of interest on chromosome 7 and 18. Therefore, a significantly associated locus may have been overlooked. Further systematic exploration of the loci of interest generated in both the linkage analysis and the genome-wide association study is now required. To do this, whole exome sequencing of family members is underway.

## Conclusions

This linkage study has implicated a number of novel chromosomal regions in the pathogenesis of AAD in multiplex AAD families. However, these regions are large; neither the genome-wide association analysis nor the regional case-control association results have provided substantive evidence to confirm a new AAD susceptibility gene. Nevertheless, the *NFATC1* gene is a promising locus and is certainly worthy of future investigation.

## Supporting Information

S1 Table Replication study Sequenom primer details. (DOCX)

S2 Table Details of quality control measures used in the study. (DOCX)

S3 Table Genes contained within the linked regions. (DOCX)

S4 Table Replication study results. (DOCX)
