# Correction: Linkage Analysis in Autoimmune Addison’s Disease: NFATC1 as a Potential Novel Susceptibility Locus

**DOI:** 10.1371/journal.pone.0138844

**Published:** 2015-09-18

**Authors:** Anna L. Mitchell, Anette Bøe Wolff, Katie MacArthur, Jolanta U. Weaver, Bijay Vaidya, The Swedish Addison Registry Study Group, Martina M. Erichsen, Rebecca Darlay, Eystein S. Husebye, Heather J. Cordell, Simon H. S. Pearce

The sixth author’s name is incorrect. The correct name is: The Swedish Addision Registry Study Group. The correct citation is: Mitchell AL, Bøe Wolff A, MacArthur K, Weaver JU, Vaidya B, The Swedish Addison Registry Study Group, et al. (2015) Linkage Analysis in Autoimmune Addison’s Disease: NFATC1 as a Potential Novel Susceptibility Locus. PLoS ONE 10(6): e0123550. doi:10.1371/journal.pone.0123550.
