# Supplementary material for: Linkage Analysis in Autoimmune Addison’s Disease: NFATC1 as a Potential Novel Susceptibility Locus
Source: PLoS One. 2015 Jun 4;10(6):e0123550. doi: 10.1371/journal.pone.0123550 (PMC4456164; doi:10.1371/journal.pone.0123550)
Supplement: S1 Table — (DOCX) [file pone.0123550.s001.docx]

Supplementary table S1: Sequenom primer sequences for the replication study.

| **SNP ID** | **Primer 1** | **Primer 2** | **Extension primer** |
| --- | --- | --- | --- |
| rs7243052 | ACGTTGGATGTATGACTGGCAAAACCTCCC | ACGTTGGATGGGCCTTCAATGTATTGACTC | CCCATCCCCAAACACAC |
| rs7236339 | ACGTTGGATGCAAGTGGACAGTTCAGTAGC | ACGTTGGATGCAAAAAGTGGTGATGCCTGC | CATGAATTCCAACCGCC |
| rs9963498 | ACGTTGGATGTCAGCCTCAGATTTGAGCAG | ACGTTGGATGTTGAATTGCAAAGCCTTCTC | GCAGGGCTGACTCATTT |
| rs2277714 | ACGTTGGATGCGTATGAGCTTCGGATTGAG | ACGTTGGATGTGCCCTCCGTCTCGTAGTG | GAGGTGCAGCCCAAGTCC |
| rs4799055 | ACGTTGGATGACAGCGGTTTCTCAAATCAC | ACGTTGGATGATCCAGGACAGGAGTCTTTG | TCTCAAATCACCTAAAGGG |
| rs12964699 | ACGTTGGATGGTACAGAAGCACTGTGGGAG | ACGTTGGATGGTGTAAGTGTATCCGCATCC | AGCACTGTGGGAGTTAGCA |
| rs8091998 | ACGTTGGATGCCACTTAGCCAGGGACTCT | ACGTTGGATGTAAGAATCAATTCCAGGCCC | aAGGGGTCCCAGGTGGTGG |
| rs11875940 | ACGTTGGATGTGTCTCCGCTTCTCTTTCCA | ACGTTGGATGGCACTTAATGAACACCTACC | CCCTTTTGGGTTTAGCACCG |
| rs2974285 | ACGTTGGATGCACCATGCTGCAGACACAC | ACGTTGGATGAGAGAAACCCTTCAGCTGTG | ccgaTGCAGACACACCCCCGC |
| rs2067534 | ACGTTGGATGCTGTGTGGAATCACTGGCAT | ACGTTGGATGCACAGCCTTAATATTTTATC | gtACTGGCATTTTAAAGTTCT |
| rs6506866 | ACGTTGGATGGCTGATTCAATCTGAAGGTG | ACGTTGGATGGGAAAGAAAAGCTATAATGG | TCAATCTGAAGGTGAGTATTG |
| rs1064059 | ACGTTGGATGCATCTCAGCCCAAAGTTGTG | ACGTTGGATGGTGTAGTTCTGGACTTTCCG | ctacCTTCAGAATGTTCCATGC |
| rs12455687 | ACGTTGGATGTAGGAAGCCAAGGGCGTGT | ACGTTGGATGTCAGGCTGCCAAAGCGTGAG | ggggCAGGGGAGCGTATCCCGA |
| rs7228520 | ACGTTGGATGAGAATGGCTGATTTGATCCC | ACGTTGGATGGGAATTTGCATCTTACAGGC | ccccATTTGATCCCTGGAATAGA |
| rs8095791 | ACGTTGGATGGCCTTGGTTCCTGAATTCAC | ACGTTGGATGTGTGGAATCACCCTCCAGCA | TTCACACTTAAAAAGAGAAACGA |
| rs2002842 | ACGTTGGATGCACTGCATCCTTCCCAGTTG | ACGTTGGATGTCATCTGCCCTATTCATGCC | ggggCTTCCCAGTTGCAGGTGAG |
| rs1960120 | ACGTTGGATGCAAGGATAAAGAAATCAGTGC | ACGTTGGATGACACATTCCGATCCCCTACA | aaagTCAGTGCATAGCACACGTAT |
| rs2015066 | ACGTTGGATGACTGCTGACATGAGAGCTGA | ACGTTGGATGCGAGGCTTGACTAGCATATC | gtagtATGTTGGGTCTGATAGGAA |
| rs7231100 | ACGTTGGATGGAAATCACAGCAGGGCTAAC | ACGTTGGATGCTATGTACACAACATGGTGC | aGCTAACATTTATTGAGAATTTACC |
| rs2085985 | ACGTTGGATGCTCCAAAGTTAGCAGAAGAG | ACGTTGGATGCTTTATGTGAATTTTTGGGC | AGAAGAAATAACTAAAATCAGAGAG |
| rs1051978 | ACGTTGGATGTGTGGAGGTCTGAAGGTTGT | ACGTTGGATGATCCTCCAACGTCAGCCCC | ggGGAGGTCTGAAGGTTGTGGCACG |
| rs8092008 | ACGTTGGATGACCGGTAAAGTTACGCTTTC | ACGTTGGATGACCTAGGAGTGGAAATATGG | ccATGTTTAATCAATCATGATGGAGA |
| rs2015612 | ACGTTGGATGCTGAGTTGTCTGTCTGATCG | ACGTTGGATGCAAGTAAAAAGGCAGACAAG | atgagCGTTGAGTTGAAGATATGTAC |
| rs6506869 | ACGTTGGATGTGGTTGTTCTCCTTTTCCTC | ACGTTGGATGAGTGGCAAAGAATCTCCGAC | CCTTTTCCTCTTCCTCC |
| rs605586 | ACGTTGGATGTCCAAGCTCTGGGACCAGT | ACGTTGGATGTTCCCCACTTCCCTAGCAAA | CAAAGAACCCACCGCAG |
| rs10237317 | ACGTTGGATGTATGGGCCACCACACCCAG | ACGTTGGATGTGGTTCTTGGCTTTCATCAG | CACACCCAGCCTCAACTA |
| rs2103187 | ACGTTGGATGATTCATGAGACCAGCCACAG | ACGTTGGATGTGGACTTCTGCTTAACATGG | TGGACTGGCACAAATTAC |
| rs11878115 | ACGTTGGATGGTTTTTAATCTCACATCCC | ACGTTGGATGGAAATCATGAATGCCGCAAC | tgCTCACATCCCACATCCC |
| rs8098006 | ACGTTGGATGCCATTTTGGCACCTAAGTTC | ACGTTGGATGACGACTAATCAGGAAGACGC | CACCTAAGTTCTTGCCTTT |
| rs372741 | ACGTTGGATGTTTGGCACCCACATATGCAG | ACGTTGGATGTGAGTTCCGGCTGGTGGAGA | gaaATATGCAGATGTCCACC |
| rs620269 | ACGTTGGATGTTGTGAGAGAGGGATTCCTG | ACGTTGGATGTCTCCTCAAGCCTGCCTATA | GGGATTCCTGAAAAAGAAAA |
| rs845056 | ACGTTGGATGTCTGGTGAATTCTGGAGAGC | ACGTTGGATGGTCTTTCCACTGTGTTTTGC | gCTCATATCCTTGAGTCCTTC |
| rs4717599 | ACGTTGGATGTTGGATCACACCCCACACG | ACGTTGGATGATCCTATAAAGCAAGGAGGG | gggtTGTTGTGTGCATACACT |
| rs2066984 | ACGTTGGATGAGCAGCCGGGATATAAAAGC | ACGTTGGATGCAGGACAGCTATCTTCTGAC | AGCCAGGCCTGGTTCCCATCCC |
| rs38319 | ACGTTGGATGGTTTCCTGGAGTCTTGGGTT | ACGTTGGATGTCCAGATTTCTGATGTGCTC | accgtAGTCTTGGGTTACACTC |
| rs9638627 | ACGTTGGATGCTTGAACTATAACTTCGAC | ACGTTGGATGTGTGTTTGAAACAGCATG | aACAATTTATGGATGAGGAAAT |
| rs2941794 | ACGTTGGATGCTCCTTAAAGGTTCTTGCGG | ACGTTGGATGTCCTAACAACAGAGAACCTC | acgCCAGGTTTATTTTGTAGCTC |
| rs2302440 | ACGTTGGATGATATGCTGTGGGTTGCTTCG | ACGTTGGATGGGCCAGCACCTCCAAAAAC | cctctTTGCTCATTCATTCATTCA |
| rs12960174 | ACGTTGGATGGCCACAGGAGAGATTGTATC | ACGTTGGATGTAAGCAGCCTTAGAAGAGTG | ttTTGTATCTACTTCTGAGAACAC |
| rs7808818 | ACGTTGGATGAGTAGTGCAGATACTGTGGC | ACGTTGGATGATAGTCAGGTGTAGGTGTCC | tcGTGCAGATACTGTGGCTCTAGG |
| rs3826573 | ACGTTGGATGTTGAACGCTGGGACTCGTAG | ACGTTGGATGGCTCGGAACTACGTATCCC | cccccGACTCGTAGTCCCGCCATGC |
| rs660749 | ACGTTGGATGTGGAGGCCGGCTGTTCTAA | ACGTTGGATGCACAGTCGTCAGGGAAATAC | ccccaGCTGTTCTAACAGGCGCGTG |
| rs10486872 | ACGTTGGATGGGTATATCATTAAGCATATC | ACGTTGGATGCCCCCAGAGTAAAAACAGAC | AGCATATCAATAAGTAAAAATATCTC |
| rs12698902 | ACGTTGGATGAAAATGCAGGGAAGGTCAAG | ACGTTGGATGGACAAAAATACGGGTTCCCT | acaatTTGAGTTTTGTAGAGGAAGAT |
| rs12672930 | ACGTTGGATGATTGTAGGAGAAGGGTTGGC | ACGTTGGATGTTTCTGTCCTACACTCTACC | ccaacAAGGGTTGGCGAGGTTGAGGG |
| rs9966033 | ACGTTGGATGTCGTGGGCGGGAAGAAGATG | ACGTTGGATGGGGCTTTCTCCACGAAAATG | AAGAAGATGGTCCTGTC |
| rs11081569 | ACGTTGGATGAAGAATCTATATGTCCCACC | ACGTTGGATGTTTGTCTGATGTGGCCACTG | TGCTGCCCTCTAAGCTAT |
| rs9638296 | ACGTTGGATGCAAGGCATCTATCCAAGAAG | ACGTTGGATGGCTACACAGTAATTTGGTGG | AGAAGGTGATCTTGTGGG |
| rs886661 | ACGTTGGATGGTGAATACTTCCTCAAGGGC | ACGTTGGATGCACTGGAGCACCATCTAAAG | ACTTGGTAAATGACTTCAC |
| rs2230112 | ACGTTGGATGTCACTGAGGAGAGCTGGCTG | ACGTTGGATGGTTGAGGCTGTACTTCCTCT | ccaTCCAGACCCGCGTCCCC |
| rs754093 | ACGTTGGATGAGCAGCTCGCGATGCCACC | ACGTTGGATGTGGCATCAGGGTGCTTCTCT | aaGTGGCCGGCTTCCCGCCC |
| rs7228827 | ACGTTGGATGCAACCCCACTACCAGTCATT | ACGTTGGATGCTCATTCATTTGGAATTAGGG | cCCAGTCATTAATTTCTGCCC |
| rs515754 | ACGTTGGATGCTATGGAGAAGCGCACCTG | ACGTTGGATGAGTACAGCACGTCCACGTC | aTCCCCTGGCTTCGGTGTGGC |
| rs4799020 | ACGTTGGATGAACTCCAGCTTTGATTAGTG | ACGTTGGATGGGTGCCTATAAGAAAACAC | accGCTTTGATTAGTGTTGGTA |
| rs11773571 | ACGTTGGATGACAGTCCTGTCCACCAACAC | ACGTTGGATGAAAATAGGGAGTGAAGGGAG | gtaacCACCCCTGTACGACCAGA |
| rs3857700 | ACGTTGGATGGTGCGTGGCCTCAGTGGT | ACGTTGGATGCATGCACACCATGGCTGATG | gggacGGCCTCAGTGGTAGTTTT |
| rs4719155 | ACGTTGGATGAGGCCAGAAAGCTATTGTCC | ACGTTGGATGATGGGCTGAGTGTCCACATC | accccAAAGCTATTGTCCTAGCCT |
| rs12458154 | ACGTTGGATGGCAGACCAGGAACTACCTTT | ACGTTGGATGGCTAGAGGTATTCTGTGGTC | accGGAACTACCTTTAAAATCAGA |
| rs8090692 | ACGTTGGATGGGAGAGCTGAATTAGGAATG | ACGTTGGATGGCATGGTAATAGCAGGAACG | ggacgGAATTAGGAATGCTCTGTT |
| rs4799141 | ACGTTGGATGTTTGGGGATGCTGCGTTGTC | ACGTTGGATGGAGAAGGATTCTACCCACTG | cccggTGTCCCCCTTGCTGAAGCTC |
| rs10807735 | ACGTTGGATGCTGATACTGAGATCATGGGC | ACGTTGGATGAAGGGCACCAAGGTGCTTTA | cagagGTGGCTTTTCCAGCCTGTGC |
| rs12955458 | ACGTTGGATGCATAACTTGCTAATTTCAGAC | ACGTTGGATGCAGACTCTACATTATCTAC | cacacCAGACTTTCCTTCACCTCTTA |
| rs4719176 | ACGTTGGATGTTTTCTTTCCTGTGTTTCC | ACGTTGGATGCCAGTGTTAAATTTTGTCCC | ttcCCTGTGTTTCCATTAAAATAATC |
| rs9959454 | ACGTTGGATGACCGCTTATTCTGAAGATG | ACGTTGGATGCCATATCATCGTTTCCTATTG | GATAAAAATTTCAGGATAAATTACTC |
| rs38307 | ACGTTGGATGGGGTCAAAGTGCTGTCATTG | ACGTTGGATGCCAGGGTCTTGTATGGTTTG | cccagAAGAAATTATTCATCAACCTCA |
