# Supplementary material for: Linkage Analysis in Autoimmune Addison’s Disease: NFATC1 as a Potential Novel Susceptibility Locus
Source: PLoS One. 2015 Jun 4;10(6):e0123550. doi: 10.1371/journal.pone.0123550 (PMC4456164; doi:10.1371/journal.pone.0123550)
Supplement: S2 Table — (DOCX) [file pone.0123550.s002.docx]

**Supplementary table S1: details of quality control measures used in the study**

| **Study** | **QC measure** | **Number of SNPs / individuals excluded** | Total inclusions |
| --- | --- | --- | --- |
| **Linkage study – pedigree QC** | Individual gender check | 1 family (3 individuals) |  |
|  | Individual levels of heterozygosity | 0 individuals |  |
|  | Allele sharing check | 0 individuals |  |
|  | Individual missingness (genotyping call rate <97.5% in any individual) | 1 individual |  |
|  | Familial Mendelian error rate >5% | 0 families | 117 individuals in 23 families |
| **Linkage and genome-wide association study – genotyping QC** | Locus missingness (SNP genotyping call rate <99%) | 80,150 SNPs |  |
|  | Locus Mendelian error rate >10% | 2 SNPs |  |
| **Linkage study – marker thinning** | Linkage disequilibrium calculation (r^2^ <0.3) and exclusion of SNPS with MAF <0.4 | 792,695 SNPs |  |
|  | Arbitrary thinning to 4 SNPs per cM | 22,004 SNPs | 14,771 SNPs |
| **Genome-wide association study – marker selection** | MAF <0.05 or out of HWE (P <1.0 x 10^-8^), | 234,352 SNPs | 595,118 SNPs |
| **Replication study – genotyping QC** | Locus missingness (SNP genotyping call rate <95%) | 3 SNPs |  |
|  | Out of HWE in the control cohort (P <0.01) | 1 SNP | 60 SNPs |

Footnote: The Affymetrix SNP 6.0 array contains 909,622 markers in total. HWE – Hardy Weinberg Equilibrium. MAF – minor allele frequency. Gender check - individuals whose genetic sex is discordant to their phenotypic gender (due to errors in sample handling). Heterozygosity - heterozygosity rates were calculated for each individual to estimate deviation from HWE suggesting sample contamination, true inbreeding or population stratification. The mean calculated heterozygosity rate was 0.32 and there were no outliers (minimum 0.31, maximum 0.35). Allele sharing check - a measure of allele sharing in unrelated and related individuals was carried out to ensure that that there was no excess identical by descent (IBD) allele sharing in unrelated individuals, and that related individuals shared an appropriate proportion of alleles IBD. Independent SNPs not in linkage disequilibrium (LD) (r^2^ <0.3) were then selected to avoid false positive linkage results. To do this, the WTCCC 1958 birth cohort genome-wide data were used to calculate LD and then select independent SNPs. Heterozygous SNPs are most informative for linkage analysis, allowing allele flow through pedigrees to be followed, therefore SNPs with a MAF of less than 0.4 were excluded. Selected markers were arbitrarily thinned to 4 SNPs per centimorgan (cM) using MapThin.
