# Supplementary material for: Linkage Analysis in Autoimmune Addison’s Disease: NFATC1 as a Potential Novel Susceptibility Locus
Source: PLoS One. 2015 Jun 4;10(6):e0123550. doi: 10.1371/journal.pone.0123550 (PMC4456164; doi:10.1371/journal.pone.0123550)
Supplement: S3 Table — (DOCX) [file pone.0123550.s003.docx]

Supplementary table S2: Genes contained within the linked regions.

Genes in the observed linked regions, as listed in the HapMap database. Linkage peak positions are given in both centimorgan (cM) and in base pairs (bp). The gene at the position of maximum linkage is indicated with underlined characters. Where the position of maximum linkage detected is between two genes, both genes are underlined. The single nucleotide polymorphism (SNP) where maximum linkage was observed is shown by rs identification number and bp position. A peak LOD score is given.

| **PARAMETRIC ANALYSIS, DOMINANT MODEL** | | | | |
| --- | --- | --- | --- | --- |
| **Chromosome (cM position; bp position)** | **Genes** | | **Maximum linkage (rs ID; bp position)** | **LOD/HLOD** |
| Chr 18 (116.5 – 121.9 cM; 75241668 – 77950543 bp) | *SALL3* | | *rs1113678*; 76554812 bp | 3.0/3.0 |
|  | *ATP9B* | |  |  |
|  | *NFATC1* | |  |  |
|  | *CTDP1* | |  |  |
|  | *KCNG2* | |  |  |
|  | *PQLC1* | |  |  |
|  | *TXNL4A* | |  |  |
|  | *ZNF508* | |  |  |
|  | *PARD6G* | |  |  |
| Chr 9 (36– 40.4 cM; 17486802 – 19751149 bp) | *LOC54875* | |  |  |
|  | *SH3GL2* | |  |  |
|  | *ADAMTSL1* | |  |  |
|  | *LOC158297* | | *rs10123624*; 19025385 bp | 2.9/2.9 |
|  | *RRAGA* | |  |  |
|  | *LOC54801* | |  |  |
|  | *ADFP* | |  |  |
|  | *DENND43* | |  |  |
|  | *RPS6* | |  |  |
|  | *ASAH3L* | |  |  |
|  | *SLC24A2* | |  |  |
| Chr 7 (82.4 – 86.2 cM; 70020160 – 73809454 bp) | *AUTS2* | | *rs10263367*; 70082089 bp | 2.88/2.88 |
|  | *WBSCR17* | |  |  |
|  | *CALN1* | |  |  |
|  | *LOC441251* | |  |  |
|  | *POM 121* | |  |  |
|  | *NSUN5C* | |  |  |
|  | *TRIM74* | |  |  |
|  | *LOC 541473* | |  |  |
|  | *FKBP6* | |  |  |
|  | *LOC442582* | |  |  |
|  | *LOC 442578* | |  |  |
|  | *LOC 441257* | |  |  |
|  | *LOC 389517* | |  |  |
|  | *NSUN5* | |  |  |
|  | *TRIM50* | |  |  |
|  | *FZD9* | |  |  |
|  | *BAZ1B* | |  |  |
|  | *TBL2* | |  |  |
|  | *MLXIPL* | |  |  |
|  | *VPS37D* | |  |  |
|  | *WBSCR18 / 22 / 27 / 29* | |  |  |
|  | *STX1A* | |  |  |
|  | *ABHD11* | |  |  |
|  | *CLDN3* | |  |  |
|  | *ELN* | |  |  |
|  | *LIMK1* | |  |  |
|  | *EIF4H* | |  |  |
|  | *LAT2* | |  |  |
|  | *RFC2* | |  |  |
|  | *CLIP2* | |  |  |
| Chr 7 (69.8 – 71.7 cM; 47565504 – 49876993 bp) | *TNS3* | |  |  |
|  | *PKD1L1* | |  |  |
|  | *FLJ21075* | |  |  |
|  | *SUNC1* | |  |  |
|  | *HUS1* | |  |  |
|  | *UPP1* | |  |  |
|  | *ABCA13* | | *rs13228770*; 49457067 bp | 1.28/2.09 |
|  | *VWC2* | |  |  |
| **NON-PARAMETRIC ANALYSIS** | | | | |
| **Chromosome (cM position; bp position)** | | **Genes** | **Maximum linkage (rs ID; bp position)** | **Linear LOD/exponential LOD** |
| Chr 6 (46.0 – 55.4 cM; 22375648 – 35968100 bp)– linear LOD 3.13 | | *HDGFL1* |  |  |
|  | | *NRSN1* |  |  |
|  | | *DCDC2* |  |  |
|  | | *KAAG1* |  |  |
|  | | *MRS2* |  |  |
|  | | *GPLD1* |  |  |
|  | | *ALDH5A1* |  |  |
|  | | *KIAA0319* |  |  |
|  | | *TTRAP* |  |  |
|  | | *THEM2* |  |  |
|  | | *C6orf62* |  |  |
|  | | *GMNN* |  |  |
|  | | *C6orf32* |  |  |
|  | | *LRRC16* |  |  |
|  | | *SCGN* |  |  |
|  | | *HIST1H* |  |  |
|  | | *SLC17A4* |  |  |
|  | | *TRIM38* |  |  |
|  | | *HFE* |  |  |
|  | | *BTN* |  |  |
|  | | *HMGN4* |  |  |
|  | | *ABT1* |  |  |
|  | | *ZNF* |  |  |
|  | | *GUSBL1* |  |  |
|  | | *LOC346157* |  |  |
|  | | *OR* |  |  |
|  | | *PGBD1* |  |  |
|  | | *GPX6* |  |  |
|  | | *TRIM 27* |  |  |
|  | | *LOC651503* |  |  |
|  | | *MAS1L* |  |  |
|  | | *GABBR1* |  |  |
|  | | *MOG* |  |  |
|  | | *HLA-F* |  |  |
|  | | *HLA-G* |  |  |
|  | | *HLA-A* |  |  |
|  | | *HCG9* |  |  |
|  | | *ZNRD1* |  |  |
|  | | *PPP1R11* |  |  |
|  | | *RNF39* |  |  |
|  | | *FLJ45422* |  |  |
|  | | *RPP21* |  |  |
|  | | *HLA-E* |  |  |
|  | | *GNL1* |  |  |
|  | | *PRR3* |  |  |
|  | | *ABCF1* |  |  |
|  | | *PPP1R10* |  |  |
|  | | *MRPS18B* |  |  |
|  | | *DHX16* |  |  |
|  | | *NRM* |  |  |
|  | | *TUBB* |  |  |
|  | | *MDC1* |  |  |
|  | | *FLOT1* |  |  |
|  | | *IER3* |  |  |
|  | | *DDR1* |  |  |
|  | | *GTF2H4* |  |  |
|  | | *VARSL* |  |  |
|  | | *SFTPG* |  |  |
|  | | *DPCR1* |  |  |
|  | | *PSORS1C1* |  |  |
|  | | *TCF19* |  |  |
|  | | *CDSN* |  |  |
|  | | *CCHCR1* |  |  |
|  | | *POU5F1* |  |  |
|  | | *HCG27* |  |  |
|  | | *HLA-C* |  |  |
|  | | *HLA-B* |  |  |
|  | | *HCP5* |  |  |
|  | | *MICB* |  |  |
|  | | *BAT1-5* |  |  |
|  | | *ATP6V1GZ* |  |  |
|  | | *NFKB1L1* |  |  |
|  | | *TNF alpha* |  |  |
|  | | *LTB* |  |  |
|  | | *LTA* |  |  |
|  | | *LST1* |  |  |
|  | | *NCR3* |  |  |
|  | | *AIF1* |  |  |
|  | | *APOM* |  |  |
|  | | *CSNK2B* |  |  |
|  | | *LY6G5B* |  |  |
|  | | *DDAH2* |  |  |
|  | | *CLIC1* |  |  |
|  | | *MSH5* |  |  |
|  | | *VARS* |  |  |
|  | | *LSM2* |  |  |
|  | | *HSPA1L* |  |  |
|  | | *NEU1* |  |  |
|  | | *SLC44A4* |  |  |
|  | | *EHMT2* |  |  |
|  | | *ZBTB12* |  |  |
|  | | *C2* |  |  |
|  | | *CFB* | *rs2072633;* 31919578 bp | 3.13/3.01 |
|  | | *RDBP* |  |  |
|  | | *SKIV2C* |  |  |
|  | | *DOM3Z* |  |  |
|  | | *STK19* |  |  |
|  | | *C4B* |  |  |
|  | | *C4A* |  |  |
|  | | *CYP21A2* |  |  |
|  | | *TNXB* |  |  |
|  | | *CREBL1* |  |  |
|  | | *FKBPL* |  |  |
|  | | *PRRT1* |  |  |
|  | | *PPT2* |  |  |
|  | | *EGFL8* |  |  |
|  | | *AGPAT1* |  |  |
|  | | *RNF5* |  |  |
|  | | *AGER* |  |  |
|  | | *GPSM3* |  |  |
|  | | *NOTCH4* |  |  |
|  | | *PBX2* |  |  |
|  | | *LOC401252* |  |  |
|  | | *BTNL2* |  |  |
|  | | *HLA-DRA* |  |  |
|  | | *HLA-DRB5* |  |  |
|  | | *HLA-DRB1* |  |  |
|  | | *HLA-DQA1* |  |  |
|  | | *HLA-DQA2* |  |  |
|  | | *HLA-DOB* |  |  |
|  | | *PSMB8* |  |  |
|  | | *TAP2 /1* |  |  |
|  | | *HLA-DMB* |  |  |
|  | | *HLA-DMA* |  |  |
|  | | *BRD2* |  |  |
|  | | *HLA-DOA* |  |  |
|  | | *HLA-DPA1* |  |  |
|  | | *HLA-DPB1* |  |  |
|  | | *COL11A2* |  |  |
|  | | *RXRB* |  |  |
|  | | *SLC39A7* |  |  |
|  | | *HSD17B8* |  |  |
|  | | *RING 1* |  |  |
|  | | *VPS52* |  |  |
|  | | *RPS18* |  |  |
|  | | *B3GALT4* |  |  |
|  | | *WDR46* |  |  |
|  | | *RGL2* |  |  |
|  | | *PFDN6* |  |  |
|  | | *TAPBP* |  |  |
|  | | *DAXX* |  |  |
|  | | *KIFC1* |  |  |
|  | | *PHF1* |  |  |
|  | | *CUTA* |  |  |
|  | | *ZBTB9* |  |  |
|  | | *SYNGAP1* |  |  |
|  | | *BAK1* |  |  |
|  | | *ITPR3* |  |  |
|  | | *IHPK3* |  |  |
|  | | *LEMD2* |  |  |
|  | | *MLN* |  |  |
|  | | *GRM4* |  |  |
|  | | *HMGA1* |  |  |
|  | | *RPS10* |  |  |
|  | | *PACSIN1* |  |  |
|  | | *SPDEF* |  |  |
|  | | *SNPPC* |  |  |
|  | | *TAF11* |  |  |
|  | | *ANKS1A* |  |  |
|  | | *TCP11* |  |  |
|  | | *SCUBE3* |  |  |
|  | | *ZNF76* |  |  |
|  | | *DEF6* |  |  |
|  | | *PPARD* |  |  |
|  | | *FANCE* |  |  |
|  | | *RPL10A* |  |  |
|  | | *TEAD3* |  |  |
|  | | *TULP1* |  |  |
|  | | *FKBP5* |  |  |
|  | | *CLPS* |  |  |
|  | | *LHFPL5* |  |  |
|  | | *SRPK1* |  |  |
|  | | *SLC26A8* |  |  |
