# Supplementary material for: Linkage Analysis in Autoimmune Addison’s Disease: NFATC1 as a Potential Novel Susceptibility Locus
Source: PLoS One. 2015 Jun 4;10(6):e0123550. doi: 10.1371/journal.pone.0123550 (PMC4456164; doi:10.1371/journal.pone.0123550)
Supplement: S4 Table — (DOCX) [file pone.0123550.s004.docx]

Supplementary table S4: replication study results.

|  | | **Hapmap CEU n=113** | | **Norway AAD**  **n=384** | | **Norway controls n=384** | |  | **Sweden AAD n=367** | | **Sweden controls n=366** | |  | **UK AAD n=346** | | **UK controls n=367** | |  | **Meta-analysis** | |
| --- | --- | --- | --- | --- | --- | --- | --- | --- | --- | --- | --- | --- | --- | --- | --- | --- | --- | --- | --- | --- |
|  |  |  |  |  |  |  |  |  | Sweden  AAD  n=345 | | Sweden  controls  n=344 | |  |  |  |  |  |  |  |  |
| **Chr: position** |  | **counts** | **allele freq** | **counts** | **allele freq** | **counts** | **allele freq** | **P**  **value** | **counts** | **allele freq** | **counts** | **allele freq** | **P**  **value** | **counts** | **allele freq** | **counts** | **allele freq** | **P**  **value** | **P value** | **I^2^ (%)** |
| **Chr 18: 74,456,072** | **rs7231100** |  |  |  |  |  |  |  |  |  |  |  |  |  |  |  |  |  |  |  |
|  | **Genotype** |  |  |  |  |  |  |  |  |  |  |  |  |  |  |  |  |  |  |  |
|  | **CC** | 59 |  | 216 |  | 196 |  |  | 197 |  | 173 |  |  | 173 |  | 197 |  |  | N/S |  |
|  | **TC** | 49 |  | 146 |  | 152 |  |  | 121 |  | 133 |  |  | 148 |  | 144 |  |  |  |  |
|  | **TT** | 5 |  | 19 |  | 36 |  | 0.042 | 25 |  | 34 |  | 0.18 | 22 |  | 25 |  | 0.59 |  |  |
|  | **Allele** |  |  |  |  |  |  |  |  |  |  |  |  |  |  |  |  |  |  |  |
|  | **C** | 167 | 0.74 | 578 | 0.76 | 544 | 0.71 |  | 515 | 0.75 | 479 | 0.70 |  | 494 | 0.72 | 538 | 0.73 |  |  |  |
|  | **T** | 59 | 0.26 | 184 | 0.24 | 224 | 0.29 | 0.026 | 171 | 0.25 | 201 | 0.30 | 0.055 | 192 | 0.28 | 194 | 0.27 | 0.53 |  |  |
|  | **Call rate (%)** |  |  | 99.2 |  | 100 |  |  | 99.4 |  | 98.8 |  |  | 99.1 |  | 99.7 |  |  |  |  |
|  | **HWE (P value)** |  |  | 0.37 |  | 0.41 |  |  | 0.29 |  | 0.26 |  |  | 0.19 |  | 0.85 |  |  |  |  |
|  |  |  |  |  |  |  |  |  |  |  |  |  |  |  |  |  |  |  |  |  |
| **Chr 18: 74,498,350** | **rs11878115** |  |  |  |  |  |  |  |  |  |  |  |  |  |  |  |  |  |  |  |
|  | **Genotype** |  |  |  |  |  |  |  |  |  |  |  |  |  |  |  |  |  |  |  |
|  | **GG** | 55 |  | 216 |  | 232 |  |  | 194 |  | 212 |  |  | 180 |  | 204 |  |  | N/S |  |
|  | **AG** | 50 |  | 143 |  | 122 |  |  | 152 |  | 127 |  |  | 143 |  | 139 |  |  |  |  |
|  | **AA** | 7 |  | 22 |  | 29 |  | 0.2 | 21 |  | 25 |  | 0.19 | 22 |  | 24 |  | 0.62 |  |  |
|  | **Allele** |  |  |  |  |  |  |  |  |  |  |  |  |  |  |  |  |  |  |  |
|  | **G** | 160 | 0.71 | 575 | 0.75 | 586 | 0.77 |  | 540 | 0.74 | 551 | 0.76 |  | 503 | 0.73 | 547 | 0.75 |  |  |  |
|  | **A** | 64 | 0.29 | 187 | 0.25 | 180 | 0.23 | 0.63 | 194 | 0.26 | 177 | 0.24 | 0.35 | 187 | 0.27 | 187 | 0.25 | 0.49 |  |  |
|  | **Call rate (%)** |  |  | 99.2 |  | 99.7 |  |  | 100 |  | 99.5 |  |  | 99.7 |  | 100 |  |  |  |  |
|  | **HWE (P value)** |  |  | 0.79 |  | 0.026 |  |  | 0.21 |  | 0.32 |  |  | 0.36 |  | 0.96 |  |  |  |  |

|  |  |  |  |  |  |  |  |  |  |  |  |  |  |  |  |  |  |  |  |  |
| --- | --- | --- | --- | --- | --- | --- | --- | --- | --- | --- | --- | --- | --- | --- | --- | --- | --- | --- | --- | --- |
| **Chr 18:74,510,585** | **rs2002842** |  |  |  |  |  |  |  |  |  |  |  |  |  |  |  |  |  |  |  |
|  | **Genotype** |  |  |  |  |  |  |  |  |  |  |  |  |  |  |  |  |  |  |  |
|  | **CC** | 26 |  | 132 |  | 121 |  |  | 110 |  | 106 |  |  | 109 |  | 120 |  |  | N/S |  |
|  | **CA** | 65 |  | 170 |  | 194 |  |  | 177 |  | 177 |  |  | 176 |  | 181 |  |  |  |  |
|  | **AA** | 17 |  | 78 |  | 68 |  | 0.25 | 54 |  | 58 |  | 0.9 | 56 |  | 66 |  | 0.79 |  |  |
|  | **Allele** |  |  |  |  |  |  |  |  |  |  |  |  |  |  |  |  |  |  |  |
|  | **C** | 117 | 0.54 | 434 | 0.57 | 436 | 0.57 |  | 397 | 0.58 | 389 | 0.57 |  | 394 | 0.58 | 421 | 0.57 |  |  |  |
|  | **A** | 99 | 0.46 | 326 | 0.43 | 330 | 0.43 | 0.94 | 285 | 0.42 | 293 | 0.43 | 0.66 | 288 | 0.42 | 313 | 0.43 | 0.87 |  |  |
|  | **Call rate (%)** |  |  | 99 |  | 99.7 |  |  | 98.8 |  | 99.1 |  |  | 98.6 |  | 100 |  |  |  |  |
|  | **HWE (P value)** |  |  | 0.091 |  | 0.52 |  |  | 0.22 |  | 0.28 |  |  | 0.29 |  | 0.88 |  |  |  |  |
|  |  |  |  |  |  |  |  |  |  |  |  |  |  |  |  |  |  |  |  |  |
| **Chr 18: 74,552,272** | **rs8092008** |  |  |  |  |  |  |  |  |  |  |  |  |  |  |  |  |  |  |  |
|  | **Genotype** |  |  |  |  |  |  |  |  |  |  |  |  |  |  |  |  |  |  |  |
|  | **CC** | 29 |  | 146 |  | 126 |  |  | 107 |  | 103 |  |  | 104 |  | 111 |  |  | N/S |  |
|  | **CA** | 54 |  | 161 |  | 187 |  |  | 166 |  | 173 |  |  | 174 |  | 179 |  |  |  |  |
|  | **AA** | 30 |  | 75 |  | 71 |  | 0.17 | 68 |  | 63 |  | 0.82 | 65 |  | 76 |  | 0.81 |  |  |
|  | **Allele** |  |  |  |  |  |  |  |  |  |  |  |  |  |  |  |  |  |  |  |
|  | **C** | 112 | 0.50 | 453 | 0.59 | 439 | 0.57 |  | 380 | 0.56 | 379 | 0.56 |  | 382 | 0.56 | 401 | 0.55 |  |  |  |
|  | **A** | 114 | 0.50 | 311 | 0.41 | 329 | 0.43 | 0.4 | 302 | 0.44 | 299 | 0.44 | 0.95 | 304 | 0.44 | 331 | 0.45 | 0.73 |  |  |
|  | **Call rate (%)** |  |  | 99.5 |  | 100 |  |  | 98.8 |  | 98.5 |  |  | 99.1 |  | 99.7 |  |  |  |  |
|  | **HWE (P value)** |  |  | 0.013 |  | 0.91 |  |  | 0.8 |  | 0.52 |  |  | 0.61 |  | 0.81 |  |  |  |  |

|  |  |  |  |  |  |  |  |  |  |  |  |  |  |  |  |  |  |  |  |  |
| --- | --- | --- | --- | --- | --- | --- | --- | --- | --- | --- | --- | --- | --- | --- | --- | --- | --- | --- | --- | --- |
| **Chr 18: 74,554,842** | **rs2941794** |  |  |  |  |  |  |  |  |  |  |  |  |  |  |  |  |  |  |  |
|  | **Genotype** |  |  |  |  |  |  |  |  |  |  |  |  |  |  |  |  |  |  |  |
|  | **AA** | 72 |  | 228 |  | 227 |  |  | 220 |  | 215 |  |  | 209 |  | 223 |  |  | N/S |  |
|  | **GA** | 34 |  | 120 |  | 138 |  |  | 134 |  | 129 |  |  | 117 |  | 124 |  |  |  |  |
|  | **GG** | 6 |  | 34 |  | 18 |  | 0.046 | 13 |  | 17 |  | 0.73 | 17 |  | 20 |  | 0.96 |  |  |
|  | **Allele** |  |  |  |  |  |  |  |  |  |  |  |  |  |  |  |  |  |  |  |
|  | **A** | 178 | 0.79 | 576 | 0.75 | 592 | 0.77 |  | 574 | 0.78 | 559 | 0.77 |  | 535 | 0.78 | 570 | 0.78 |  |  |  |
|  | **G** | 46 | 0.21 | 188 | 0.25 | 174 | 0.23 | 0.38 | 160 | 0.22 | 163 | 0.23 | 0.72 | 151 | 0.22 | 164 | 0.22 | 0.88 |  |  |
|  | **Call rate (%)** |  |  | 99.5 |  | 99.7 |  |  | 100 |  | 98.6 |  |  | 99.1 |  | 100 |  |  |  |  |
|  | **HWE (P value)** |  |  | 0.0027 |  | 0.61 |  |  | 0.17 |  | 0.67 |  |  | 0.9 |  | 0.61 |  |  |  |  |
|  |  |  |  |  |  |  |  |  |  |  |  |  |  |  |  |  |  |  |  |  |
| **Chr 18: 74,596,466** | **rs8095791** |  |  |  |  |  |  |  |  |  |  |  |  |  |  |  |  |  |  |  |
|  | **Genotype** |  |  |  |  |  |  |  |  |  |  |  |  |  |  |  |  |  |  |  |
|  | **TT** | 36 |  | 170 |  | 156 |  |  | 119 |  | 131 |  |  | 133 |  | 134 |  |  | N/S |  |
|  | **TC** | 56 |  | 161 |  | 184 |  |  | 176 |  | 147 |  |  | 159 |  | 174 |  |  |  |  |
|  | **CC** | 21 |  | 51 |  | 44 |  | 0.27 | 48 |  | 61 |  | 0.095 | 52 |  | 59 |  | 0.83 |  |  |
|  | **Allele** |  |  |  |  |  |  |  |  |  |  |  |  |  |  |  |  |  |  |  |
|  | **T** | 128 | 0.57 | 501 | 0.66 | 496 | 0.65 |  | 414 | 0.60 | 409 | 0.60 |  | 425 | 0.62 | 442 | 0.60 |  |  |  |
|  | **C** | 98 | 0.43 | 263 | 0.34 | 272 | 0.35 | 0.68 | 272 | 0.40 | 269 | 0.40 | 0.99 | 263 | 0.38 | 292 | 0.40 | 0.55 |  |  |
|  | **Call rate (%)** |  |  | 99.5 |  | 100 |  |  | 99.4 |  | 98.5 |  |  | 99.4 |  | 100 |  |  |  |  |
|  | **HWE (P value)** |  |  | 0.19 |  | 0.35 |  |  | 0.18 |  | 0.083 |  |  | 0.69 |  | 0.84 |  |  |  |  |

|  |  |  |  |  |  |  |  |  |  |  |  |  |  |  |  |  |  |  |  |  |
| --- | --- | --- | --- | --- | --- | --- | --- | --- | --- | --- | --- | --- | --- | --- | --- | --- | --- | --- | --- | --- |
| **Chr 18: 74,597,608** | **rs2015066** |  |  |  |  |  |  |  |  |  |  |  |  |  |  |  |  |  |  |  |
|  | **Genotype** |  |  |  |  |  |  |  |  |  |  |  |  |  |  |  |  |  |  |  |
|  | **TT** | 35 |  | 160 |  | 147 |  |  | 115 |  | 126 |  |  | 129 |  | 130 |  |  | N/S |  |
|  | **CT** | 57 |  | 167 |  | 188 |  |  | 175 |  | 148 |  |  | 157 |  | 174 |  |  |  |  |
|  | **CC** | 21 |  | 53 |  | 49 |  | 0.38 | 54 |  | 66 |  | 0.14 | 56 |  | 63 |  | 0.82 |  |  |
|  | **Allele** |  |  |  |  |  |  |  |  |  |  |  |  |  |  |  |  |  |  |  |
|  | **T** | 127 | 0.56 | 487 | 0.64 | 482 | 0.63 |  | 405 | 0.59 | 400 | 0.59 |  | 415 | 0.61 | 434 | 0.59 |  |  |  |
|  | **C** | 99 | 0.44 | 273 | 0.36 | 286 | 0.37 | 0.59 | 283 | 0.41 | 280 | 0.41 | 0.99 | 269 | 0.39 | 300 | 0.41 |  |  |  |
|  | **Call rate (%)** |  |  | 99 |  | 100 |  |  | 99.7 |  | 98.8 |  |  | 98.8 |  | 100 |  |  |  |  |
|  | **HWE (P value)** |  |  | 0.38 |  | 0.35 |  |  | 0.35 |  | 0.061 |  |  | 0.48 |  | 0.71 |  |  |  |  |
|  |  |  |  |  |  |  |  |  |  |  |  |  |  |  |  |  |  |  |  |  |
| **Chr 18: 74,636,915** | **rs8098006** |  |  |  |  |  |  |  |  |  |  |  |  |  |  |  |  |  |  |  |
|  | **Genotype** |  |  |  |  |  |  |  |  |  |  |  |  |  |  |  |  |  |  |  |
|  | **GG** | 48 |  | 190 |  | 193 |  |  | 168 |  | 172 |  |  | 172 |  | 195 |  |  | N/S |  |
|  | **GT** | 57 |  | 172 |  | 155 |  |  | 166 |  | 148 |  |  | 135 |  | 144 |  |  |  |  |
|  | **TT** | 7 |  | 20 |  | 35 |  | 0.082 | 33 |  | 39 |  | 0.47 | 35 |  | 27 |  | 0.38 |  |  |
|  | **Allele** |  |  |  |  |  |  |  |  |  |  |  |  |  |  |  |  |  |  |  |
|  | **G** | 157 | 0.69 | 552 | 0.72 | 541 | 0.71 |  | 502 | 0.68 | 492 | 0.69 |  | 479 | 0.70 | 534 | 0.73 |  |  |  |
|  | **T** | 71 | 0.31 | 212 | 0.28 | 225 | 0.29 | 0.48 | 232 | 0.32 | 226 | 0.31 | 0.96 | 205 | 0.30 | 198 | 0.27 | 0.22 |  |  |
|  | **Call rate (%)** |  |  | 99.5 |  | 99.7 |  |  | 100 |  | 98.1 |  |  | 98.8 |  | 99.7 |  |  |  |  |
|  | **HWE (P value)** |  |  | 0.016 |  | 0.63 |  |  | 0.38 |  | 0.4 |  |  | 0.27 |  | 0.95 |  |  |  |  |

|  |  |  |  |  |  |  |  |  |  |  |  |  |  |  |  |  |  |  |  |  |
| --- | --- | --- | --- | --- | --- | --- | --- | --- | --- | --- | --- | --- | --- | --- | --- | --- | --- | --- | --- | --- |
| **Chr 18: 74,651,916** | **rs9963498** |  |  |  |  |  |  |  |  |  |  |  |  |  |  |  |  |  |  |  |
|  | **Genotype** |  |  |  |  |  |  |  |  |  |  |  |  |  |  |  |  |  |  |  |
|  | **GG** | 92 |  | 296 |  | 307 |  |  | 276 |  | 255 |  |  | 288 |  | 295 |  |  | N/S |  |
|  | **CG** | 18 |  | 80 |  | 72 |  |  | 63 |  | 84 |  |  | 56 |  | 67 |  |  |  |  |
|  | **CC** | 2 |  | 6 |  | 5 |  | 0.7 | 4 |  | 2 |  | 0.11 | 0 |  | 5 |  | 0.07 |  |  |
|  | **Allele** |  |  |  |  |  |  |  |  |  |  |  |  |  |  |  |  |  |  |  |
|  | **G** | 202 | 0.90 | 672 | 0.88 | 686 | 0.89 |  | 615 | 0.90 | 594 | 0.87 |  | 632 | 0.92 | 657 | 0.90 |  |  |  |
|  | **C** | 22 | 0.10 | 92 | 0.12 | 82 | 0.11 | 0.4 | 71 | 0.10 | 88 | 0.13 | 0.14 | 56 | 0.08 | 77 | 0.10 | 0.13 |  |  |
|  | **Call rate (%)** |  |  | 99.5 |  | 100 |  |  | 99.4 |  | 99.1 |  |  | 99.4 |  | 100 |  |  |  |  |
|  | **HWE (P value)** |  |  | 0.82 |  | 0.74 |  |  | 0.85 |  | 0.076 |  |  | 0.1 |  | 0.59 |  |  |  |  |
|  |  |  |  |  |  |  |  |  |  |  |  |  |  |  |  |  |  |  |  |  |
| **Chr 18: 74,653,655** | **rs11875940** |  |  |  |  |  |  |  |  |  |  |  |  |  |  |  |  |  |  |  |
|  | **Genotype** |  |  |  |  |  |  |  |  |  |  |  |  |  |  |  |  |  |  |  |
|  | **CC** | 91 |  | 295 |  | 308 |  |  | 276 |  | 253 |  |  | 288 |  | 295 |  |  | N/S |  |
|  | **CA** | 18 |  | 81 |  | 71 |  |  | 63 |  | 83 |  |  | 56 |  | 67 |  |  |  |  |
|  | **AA** | 2 |  | 6 |  | 5 |  | 0.6 | 4 |  | 2 |  | 0.11 | 0 |  | 5 |  | 0.07 |  |  |
|  | **Allele** |  |  |  |  |  |  |  |  |  |  |  |  |  |  |  |  |  |  |  |
|  | **C** | 200 | 0.90 | 671 | 0.88 | 687 | 0.89 |  | 615 | 0.90 | 589 | 0.87 |  | 632 | 0.92 | 657 | 0.90 |  |  |  |
|  | **A** | 22 | 0.10 | 93 | 0.12 | 81 | 0.11 | 0.32 | 71 | 0.10 | 87 | 0.13 | 0.15 | 56 | 0.08 | 77 | 0.10 | 0.13 |  |  |
|  | **Call rate (%)** |  |  | 99.5 |  | 100 |  |  | 99.4 |  | 98.3 |  |  | 99.4 |  | 100 |  |  |  |  |
|  | **HWE (P value)** |  |  | 0.87 |  | 0.69 |  |  | 0.85 |  | 0.081 |  |  | 0.1 |  | 0.59 |  |  |  |  |

|  |  |  |  |  |  |  |  |  |  |  |  |  |  |  |  |  |  |  |  |  |
| --- | --- | --- | --- | --- | --- | --- | --- | --- | --- | --- | --- | --- | --- | --- | --- | --- | --- | --- | --- | --- |
| **Chr 18: 74,707,894** | **rs6506866** |  |  |  |  |  |  |  |  |  |  |  |  |  |  |  |  |  |  |  |
|  | **Genotype** |  |  |  |  |  |  |  |  |  |  |  |  |  |  |  |  |  |  |  |
|  | **TT** | 38 |  | 130 |  | 142 |  |  | 118 |  | 113 |  |  | 128 |  | 156 |  |  | N/S |  |
|  | **AT** | 60 |  | 190 |  | 182 |  |  | 164 |  | 164 |  |  | 166 |  | 167 |  |  |  |  |
|  | **AA** | 14 |  | 61 |  | 60 |  | 0.71 | 61 |  | 57 |  | 0.94 | 49 |  | 44 |  | 0.33 |  |  |
|  | **Allele** |  |  |  |  |  |  |  |  |  |  |  |  |  |  |  |  |  |  |  |
|  | **T** | 136 | 0.61 | 450 | 0.59 | 466 | 0.61 |  | 400 | 0.58 | 390 | 0.58 |  | 422 | 0.62 | 479 | 0.65 |  |  |  |
|  | **A** | 88 | 0.39 | 312 | 0.41 | 302 | 0.39 | 0.52 | 286 | 0.42 | 278 | 0.42 | 0.98 | 264 | 0.38 | 255 | 0.35 | 0.14 |  |  |
|  | **Call rate (%)** |  |  | 99.2 |  | 100 |  |  | 99.4 |  | 97.1 |  |  | 99.1 |  | 100 |  |  |  |  |
|  | **HWE (P value)** |  |  | 0.54 |  | 0.89 |  |  | 0.76 |  | 0.85 |  |  | 0.68 |  | 0.95 |  |  |  |  |
|  |  |  |  |  |  |  |  |  |  |  |  |  |  |  |  |  |  |  |  |  |
| **Chr 18: 74,743,955** | **rs6506869** |  |  |  |  |  |  |  |  |  |  |  |  |  |  |  |  |  |  |  |
|  | **Genotype** |  |  |  |  |  |  |  |  |  |  |  |  |  |  |  |  |  |  |  |
|  | **GG** | 46 |  | 115 |  | 98 |  |  | 112 |  | 103 |  |  | 109 |  | 145 |  |  | N/S |  |
|  | **GA** | 54 |  | 189 |  | 215 |  |  | 187 |  | 190 |  |  | 183 |  | 153 |  |  |  |  |
|  | **AA** | 13 |  | 77 |  | 70 |  | 0.19 | 68 |  | 72 |  | 0.78 | 51 |  | 67 |  | 0.0097 |  |  |
|  | **Allele** |  |  |  |  |  |  |  |  |  |  |  |  |  |  |  |  |  |  |  |
|  | **G** | 146 | 0.65 | 419 | 0.55 | 411 | 0.54 |  | 411 | 0.56 | 396 | 0.54 |  | 401 | 0.58 | 443 | 0.61 |  |  |  |
|  | **A** | 80 | 0.35 | 343 | 0.45 | 355 | 0.46 | 0.6 | 323 | 0.44 | 334 | 0.46 | 0.5 | 285 | 0.42 | 287 | 0.39 | 0.39 |  |  |
|  | **Call rate (%)** |  |  | 99.2 |  | 99.7 |  |  | 100 |  | 99.7 |  |  | 99.1 |  | 99.5 |  |  |  |  |
|  | **HWE (P value)** |  |  | 0.97 |  | 0.012 |  |  | 0.52 |  | 0.35 |  |  | 0.068 |  | 0.02 |  |  |  |  |

|  |  |  |  |  |  |  |  |  |  |  |  |  |  |  |  |  |  |  |  |  |
| --- | --- | --- | --- | --- | --- | --- | --- | --- | --- | --- | --- | --- | --- | --- | --- | --- | --- | --- | --- | --- |
| **Chr 18: 74,748,072** | **rs2085985** |  |  |  |  |  |  |  |  |  |  |  |  |  |  |  |  |  |  |  |
|  | **Genotype** |  |  |  |  |  |  |  |  |  |  |  |  |  |  |  |  |  |  |  |
|  | **AA** | 45 |  | 116 |  | 99 |  |  | 104 |  | 99 |  |  | 110 |  | 146 |  |  | N/S |  |
|  | **AG** | 54 |  | 188 |  | 215 |  |  | 178 |  | 169 |  |  | 181 |  | 154 |  |  |  |  |
|  | **GG** | 12 |  | 77 |  | 70 |  | 0.18 | 61 |  | 68 |  | 0.72 | 51 |  | 67 |  | 0.014 |  |  |
|  | **Allele** |  |  |  |  |  |  |  |  |  |  |  |  |  |  |  |  |  |  |  |
|  | **A** | 144 | 0.65 | 420 | 0.55 | 413 | 0.54 |  | 386 | 0.56 | 367 | 0.55 |  | 401 | 0.59 | 446 | 0.61 |  |  |  |
|  | **G** | 78 | 0.35 | 342 | 0.45 | 355 | 0.46 | 0.6 | 300 | 0.44 | 305 | 0.45 | 0.54 | 283 | 0.41 | 288 | 0.39 | 0.41 |  |  |
|  | **Call rate (%)** |  |  | 99.2 |  | 100 |  |  | 99.4 |  | 97.7 |  |  | 98.8 |  | 100 |  |  |  |  |
|  | **HWE (P value)** |  |  | 0.96 |  | 0.013 |  |  | 0.31 |  | 0.79 |  |  | 0.093 |  | 0.022 |  |  |  |  |
|  |  |  |  |  |  |  |  |  |  |  |  |  |  |  |  |  |  |  |  |  |
| **Chr 18: 74,826,612** | **rs2974285** |  |  |  |  |  |  |  |  |  |  |  |  |  |  |  |  |  |  |  |
|  | **Genotype** |  |  |  |  |  |  |  |  |  |  |  |  |  |  |  |  |  |  |  |
|  | **AA** | 65 |  | 209 |  | 209 |  |  | 196 |  | 188 |  |  | 199 |  | 221 |  |  | N/S |  |
|  | **CA** | 41 |  | 151 |  | 155 |  |  | 143 |  | 142 |  |  | 128 |  | 115 |  |  |  |  |
|  | **CC** | 7 |  | 20 |  | 18 |  | 0.93 | 14 |  | 22 |  | 0.38 | 15 |  | 21 |  | 0.28 |  |  |
|  | **Allele** |  |  |  |  |  |  |  |  |  |  |  |  |  |  |  |  |  |  |  |
|  | **A** | 171 | 0.76 | 569 | 0.75 | 573 | 0.75 |  | 535 | 0.76 | 518 | 0.74 |  | 526 | 0.77 | 557 | 0.78 |  |  |  |
|  | **C** | 55 | 0.24 | 191 | 0.25 | 191 | 0.25 | 0.95 | 171 | 0.24 | 186 | 0.26 | 0.34 | 158 | 0.23 | 157 | 0.22 | 0.62 |  |  |
|  | **Call rate (%)** |  |  | 99 |  | 99.5 |  |  | 96.2 |  | 96.2 |  |  | 98.8 |  | 97.3 |  |  |  |  |
|  | **HWE (P value)** |  |  | 0.28 |  | 0.11 |  |  | 0.052 |  | 0.48 |  |  | 0.32 |  | 0.25 |  |  |  |  |

|  |  |  |  |  |  |  |  |  |  |  |  |  |  |  |  |  |  |  |  |  |
| --- | --- | --- | --- | --- | --- | --- | --- | --- | --- | --- | --- | --- | --- | --- | --- | --- | --- | --- | --- | --- |
| **Chr 18: 74,871,808** | **rs9959454** |  |  |  |  |  |  |  |  |  |  |  |  |  |  |  |  |  |  |  |
|  | **Genotype** |  |  |  |  |  |  |  |  |  |  |  |  |  |  |  |  |  |  |  |
|  | **AA** | 66 |  | 205 |  | 224 |  |  | 205 |  | 203 |  |  | 175 |  | 182 |  |  | N/S |  |
|  | **GA** | 42 |  | 149 |  | 128 |  |  | 134 |  | 133 |  |  | 142 |  | 160 |  |  |  |  |
|  | **GG** | 4 |  | 28 |  | 31 |  | 0.27 | 24 |  | 19 |  | 0.78 | 26 |  | 23 |  | 0.7 |  |  |
|  | **Allele** |  |  |  |  |  |  |  |  |  |  |  |  |  |  |  |  |  |  |  |
|  | **A** | 174 | 0.78 | 559 | 0.73 | 576 | 0.75 |  | 544 | 0.75 | 539 | 0.76 |  | 492 | 0.72 | 524 | 0.72 |  |  |  |
|  | **G** | 50 | 0.22 | 205 | 0.27 | 190 | 0.25 | 0.36 | 182 | 0.25 | 171 | 0.24 | 0.66 | 194 | 0.28 | 206 | 0.28 | 0.98 |  |  |
|  | **Call rate (%)** |  |  | 99.5 |  | 99.7 |  |  | 98.9 |  | 97 |  |  | 99.1 |  | 99.5 |  |  |  |  |
|  | **HWE (P value)** |  |  | 0.9 |  | 0.042 |  |  | 0.74 |  | 0.64 |  |  | 0.7 |  | 0.12 |  |  |  |  |
|  |  |  |  |  |  |  |  |  |  |  |  |  |  |  |  |  |  |  |  |  |
| **Chr 18: 75,001,399** | **rs7228827** |  |  |  |  |  |  |  |  |  |  |  |  |  |  |  |  |  |  |  |
|  | **Genotype** |  |  |  |  |  |  |  |  |  |  |  |  |  |  |  |  |  |  |  |
|  | **CC** | 71 |  | 214 |  | 228 |  |  | 206 |  | 199 |  |  | 201 |  | 225 |  |  | N/S |  |
|  | **TC** | 31 |  | 152 |  | 132 |  |  | 130 |  | 141 |  |  | 125 |  | 121 |  |  |  |  |
|  | **TT** | 11 |  | 16 |  | 24 |  | 0.18 | 27 |  | 19 |  | 0.38 | 16 |  | 19 |  | 0.63 |  |  |
|  | **Allele** |  |  |  |  |  |  |  |  |  |  |  |  |  |  |  |  |  |  |  |
|  | **C** | 173 | 0.77 | 580 | 0.76 | 588 | 0.77 |  | 542 | 0.75 | 539 | 0.75 |  | 527 | 0.77 | 571 | 0.78 |  |  |  |
|  | **T** | 53 | 0.23 | 184 | 0.24 | 180 | 0.23 | 0.77 | 184 | 0.25 | 179 | 0.25 | 0.86 | 157 | 0.23 | 159 | 0.22 | 0.6 |  |  |
|  | **Call rate (%)** |  |  | 99.5 |  | 100 |  |  | 98.9 |  | 98.1 |  |  | 98.8 |  | 99.5 |  |  |  |  |
|  | **HWE (P value)** |  |  | 0.085 |  | 0.41 |  |  | 0.31 |  | 0.35 |  |  | 0.54 |  | 0.6 |  |  |  |  |

|  |  |  |  |  |  |  |  |  |  |  |  |  |  |  |  |  |  |  |  |  |
| --- | --- | --- | --- | --- | --- | --- | --- | --- | --- | --- | --- | --- | --- | --- | --- | --- | --- | --- | --- | --- |
| **Chr 18: 75,059,317** | **rs4799020** |  |  |  |  |  |  |  |  |  |  |  |  |  |  |  |  |  |  |  |
|  | **Genotype** |  |  |  |  |  |  |  |  |  |  |  |  |  |  |  |  |  |  |  |
|  | **GG** | 44 |  | 121 |  | 131 |  |  | 121 |  | 114 |  |  | 102 |  | 120 |  |  | N/S |  |
|  | **GA** | 44 |  | 206 |  | 183 |  |  | 172 |  | 175 |  |  | 169 |  | 171 |  |  |  |  |
|  | **AA** | 23 |  | 55 |  | 70 |  | 0.17 | 70 |  | 68 |  | 0.9 | 70 |  | 70 |  | 0.64 |  |  |
|  | **Allele** |  |  |  |  |  |  |  |  |  |  |  |  |  |  |  |  |  |  |  |
|  | **G** | 132 | 0.59 | 448 | 0.59 | 445 | 0.58 |  | 414 | 0.57 | 403 | 0.56 |  | 373 | 0.55 | 411 | 0.57 |  |  |  |
|  | **A** | 90 | 0.41 | 316 | 0.41 | 323 | 0.42 | 0.78 | 312 | 0.43 | 311 | 0.44 | 0.82 | 309 | 0.45 | 311 | 0.43 | 0.4 |  |  |
|  | **Call rate (%)** |  |  | 99.5 |  | 100 |  |  | 98.9 |  | 97.5 |  |  | 98.6 |  | 98.4 |  |  |  |  |
|  | **HWE (P value)** |  |  | 0.029 |  | 0.66 |  |  | 0.53 |  | 0.95 |  |  | 1 |  | 0.52 |  |  |  |  |
|  |  |  |  |  |  |  |  |  |  |  |  |  |  |  |  |  |  |  |  |  |
| **Chr 18: 75,114,906** | **rs2067534** |  |  |  |  |  |  |  |  |  |  |  |  |  |  |  |  |  |  |  |
|  | **Genotype** |  |  |  |  |  |  |  |  |  |  |  |  |  |  |  |  |  |  |  |
|  | **AA** | 62 |  | 184 |  | 196 |  |  | 159 |  | 150 |  |  | 143 |  | 148 |  |  | N/S |  |
|  | **AG** | 43 |  | 160 |  | 151 |  |  | 152 |  | 146 |  |  | 158 |  | 174 |  |  |  |  |
|  | **GG** | 7 |  | 36 |  | 37 |  | 0.73 | 23 |  | 28 |  | N/A | 40 |  | 32 |  | 0.47 |  |  |
|  | **Allele** |  |  |  |  |  |  |  |  |  |  |  |  |  |  |  |  |  |  |  |
|  | **A** | 167 | 0.75 | 528 | 0.69 | 543 | 0.71 |  | 470 | 0.70 | 446 | 0.69 |  | 444 | 0.65 | 470 | 0.66 |  |  |  |
|  | **G** | 57 | 0.25 | 232 | 0.31 | 225 | 0.29 | 0.6 | 198 | 0.30 | 202 | 0.31 | N/A | 238 | 0.35 | 238 | 0.34 | 0.61 |  |  |
|  | **Call rate (%)** |  |  | 99 |  | 100 |  |  | 96.8 |  | 94.2 |  |  | 98.6 |  | 96.5 |  |  |  |  |
|  | **HWE (P value)** |  |  | 0.89 |  | 0.32 |  |  | 0.096 |  | 0.37 |  |  | 0.72 |  | 0.057 |  |  |  |  |

|  |  |  |  |  |  |  |  |  |  |  |  |  |  |  |  |  |  |  |  |  |
| --- | --- | --- | --- | --- | --- | --- | --- | --- | --- | --- | --- | --- | --- | --- | --- | --- | --- | --- | --- | --- |
| **Chr 18: 75,152,449** | **rs12955458** |  |  |  |  |  |  |  |  |  |  |  |  |  |  |  |  |  |  |  |
|  | **Genotype** |  |  |  |  |  |  |  |  |  |  |  |  |  |  |  |  |  |  |  |
|  | **AA** | 55 |  | 202 |  | 204 |  |  | 196 |  | 194 |  |  | 201 |  | 213 |  |  | N/S |  |
|  | **CA** | 53 |  | 159 |  | 141 |  |  | 143 |  | 140 |  |  | 117 |  | 136 |  |  |  |  |
|  | **CC** | 5 |  | 21 |  | 38 |  | 0.05 | 24 |  | 22 |  | 0.97 | 24 |  | 14 |  | 0.15 |  |  |
|  | **Allele** |  |  |  |  |  |  |  |  |  |  |  |  |  |  |  |  |  |  |  |
|  | **A** | 163 | 0.72 | 563 | 0.74 | 549 | 0.72 |  | 535 | 0.74 | 528 | 0.74 |  | 519 | 0.76 | 562 | 0.77 |  |  |  |
|  | **C** | 63 | 0.28 | 201 | 0.26 | 217 | 0.28 | 0.38 | 191 | 0.26 | 184 | 0.26 | 0.84 | 165 | 0.24 | 164 | 0.23 | 0.5 |  |  |
|  | **Call rate (%)** |  |  | 99.5 |  | 99.7 |  |  | 98.9 |  | 97.3 |  |  | 98.8 |  | 98.9 |  |  |  |  |
|  | **HWE (P value)** |  |  | 0.15 |  | 0.068 |  |  | 0.76 |  | 0.62 |  |  | 0.23 |  | 0.17 |  |  |  |  |
|  |  |  |  |  |  |  |  |  |  |  |  |  |  |  |  |  |  |  |  |  |
| **Chr 18: 75,187,747** | **rs12458154** |  |  |  |  |  |  |  |  |  |  |  |  |  |  |  |  |  |  |  |
|  | **Genotype** |  |  |  |  |  |  |  |  |  |  |  |  |  |  |  |  |  |  |  |
|  | **AA** | 62 |  | 206 |  | 207 |  |  | 192 |  | 188 |  |  | 171 |  | 176 |  |  | N/S |  |
|  | **CA** | 44 |  | 150 |  | 154 |  |  | 140 |  | 142 |  |  | 151 |  | 158 |  |  |  |  |
|  | **CC** | 7 |  | 26 |  | 23 |  | 0.89 | 32 |  | 30 |  | 0.95 | 22 |  | 29 |  | 0.71 |  |  |
|  | **Allele** |  |  |  |  |  |  |  |  |  |  |  |  |  |  |  |  |  |  |  |
|  | **A** | 168 | 0.74 | 562 | 0.74 | 568 | 0.74 |  | 524 | 0.72 | 518 | 0.72 |  | 493 | 0.72 | 510 | 0.70 |  |  |  |
|  | **C** | 58 | 0.26 | 202 | 0.26 | 200 | 0.26 | 0.86 | 204 | 0.28 | 202 | 0.28 | 0.99 | 195 | 0.28 | 216 | 0.30 | 0.56 |  |  |
|  | **Call rate (%)** |  |  | 99.5 |  | 100 |  |  | 99.2 |  | 98.4 |  |  | 99.4 |  | 98.9 |  |  |  |  |
|  | **HWE (P value)** |  |  | 0.85 |  | 0.42 |  |  | 0.37 |  | 0.66 |  |  | 0.13 |  | 0.43 |  |  |  |  |

|  |  |  |  |  |  |  |  |  |  |  |  |  |  |  |  |  |  |  |  |  |
| --- | --- | --- | --- | --- | --- | --- | --- | --- | --- | --- | --- | --- | --- | --- | --- | --- | --- | --- | --- | --- |
| **Chr 18: 75,213,904** | **rs2015612** |  |  |  |  |  |  |  |  |  |  |  |  |  |  |  |  |  |  |  |
|  | **Genotype** |  |  |  |  |  |  |  |  |  |  |  |  |  |  |  |  |  |  |  |
|  | **AA** | 64 |  | 211 |  | 205 |  |  | 189 |  | 188 |  |  | 158 |  | 183 |  |  | N/S |  |
|  | **CA** | 42 |  | 146 |  | 149 |  |  | 138 |  | 130 |  |  | 161 |  | 157 |  |  |  |  |
|  | **CC** | 7 |  | 25 |  | 30 |  | 0.75 | 15 |  | 21 |  | 0.54 | 25 |  | 27 |  | 0.54 |  |  |
|  | **Allele** |  |  |  |  |  |  |  |  |  |  |  |  |  |  |  |  |  |  |  |
|  | **A** | 170 | 0.75 | 568 | 0.74 | 559 | 0.73 |  | 516 | 0.75 | 506 | 0.75 |  | 477 | 0.69 | 523 | 0.71 |  |  |  |
|  | **C** | 56 | 0.25 | 196 | 0.26 | 209 | 0.27 | 0.49 | 168 | 0.25 | 172 | 0.25 | 0.73 | 211 | 0.31 | 211 | 0.29 | 0.43 |  |  |
|  | **Call rate (%)** |  |  | 99.5 |  | 100 |  |  | 99.1 |  | 98.5 |  |  | 99.4 |  | 100 |  |  |  |  |
|  | **HWE (P value)** |  |  | 0.97 |  | 0.69 |  |  | 0.1 |  | 0.81 |  |  | 0.062 |  | 0.4 |  |  |  |  |
|  |  |  |  |  |  |  |  |  |  |  |  |  |  |  |  |  |  |  |  |  |
| **Chr 18: 75,264,917** | **rs8090692** |  |  |  |  |  |  |  |  |  |  |  |  |  |  |  |  |  |  |  |
|  | **Genotype** |  |  |  |  |  |  |  |  |  |  |  |  |  |  |  |  |  |  |  |
|  | **AA** | 55 |  | 250 |  | 242 |  |  | 236 |  | 231 |  |  | 207 |  | 204 |  |  | N/S |  |
|  | **AG** | 49 |  | 121 |  | 123 |  |  | 110 |  | 118 |  |  | 116 |  | 144 |  |  |  |  |
|  | **GG** | 8 |  | 10 |  | 19 |  | 0.23 | 18 |  | 12 |  | 0.47 | 20 |  | 15 |  | 0.2 |  |  |
|  | **Allele** |  |  |  |  |  |  |  |  |  |  |  |  |  |  |  |  |  |  |  |
|  | **A** | 159 | 0.71 | 621 | 0.81 | 607 | 0.79 |  | 582 | 0.80 | 580 | 0.80 |  | 530 | 0.77 | 552 | 0.76 |  |  |  |
|  | **G** | 65 | 0.29 | 141 | 0.19 | 161 | 0.21 | 0.23 | 146 | 0.20 | 142 | 0.20 | 0.85 | 156 | 0.23 | 174 | 0.24 | 0.59 |  |  |
|  | **Call rate (%)** |  |  | 99.2 |  | 100 |  |  | 99.2 |  | 98.6 |  |  | 99.1 |  | 98.9 |  |  |  |  |
|  | **HWE (P value)** |  |  | 0.3 |  | 0.51 |  |  | 0.27 |  | 0.51 |  |  |  |  |  |  |  |  |  |

|  |  |  |  |  |  |  |  |  |  |  |  |  |  |  |  |  |  |  |  |  |
| --- | --- | --- | --- | --- | --- | --- | --- | --- | --- | --- | --- | --- | --- | --- | --- | --- | --- | --- | --- | --- |
| **Chr 18: 75,271,465** | **rs1051978** |  |  |  |  |  |  |  |  |  |  |  |  |  |  |  |  |  |  |  |
|  | **Genotype** |  |  |  |  |  |  |  |  |  |  |  |  |  |  |  |  |  |  |  |
|  | **CC** | 95 |  | 279 |  | 295 |  |  | 255 |  | 262 |  |  | 271 |  | 259 |  |  | N/S |  |
|  | **CA** | 18 |  | 93 |  | 69 |  |  | 81 |  | 72 |  |  | 66 |  | 79 |  |  |  |  |
|  | **AA** | 0 |  | 9 |  | 10 |  | 0.14 | 5 |  | 5 |  | 0.73 | 2 |  | 7 |  | N/A |  |  |
|  | **Allele** |  |  |  |  |  |  |  |  |  |  |  |  |  |  |  |  |  |  |  |
|  | **C** | 208 | 0.92 | 651 | 0.85 | 659 | 0.88 |  | 591 | 0.87 | 596 | 0.88 |  | 608 | 0.90 | 597 | 0.87 |  |  |  |
|  | **A** | 18 | 0.08 | 111 | 0.15 | 89 | 0.12 | 0.13 | 91 | 0.13 | 82 | 0.12 | 0.49 | 70 | 0.10 | 93 | 0.13 | N/A |  |  |
|  | **Call rate (%)** |  |  | 99.2 |  | 97.4 |  |  | 98.8 |  | 98.5 |  |  | 98 |  | 94 |  |  |  |  |
|  | **HWE (P value)** |  |  | 0.71 |  | 0.02 |  |  | 0.62 |  | 0.98 |  |  | 0.34 |  | 0.74 |  |  |  |  |
|  |  |  |  |  |  |  |  |  |  |  |  |  |  |  |  |  |  |  |  |  |
| **Chr 18: 75,272,049** | **rs2230112** |  |  |  |  |  |  |  |  |  |  |  |  |  |  |  |  |  |  |  |
|  | **Genotype** |  |  |  |  |  |  |  |  |  |  |  |  |  |  |  |  |  |  |  |
|  | **GG** | 42 |  | 209 |  | 202 |  |  | 174 |  | 168 |  |  | 152 |  | 173 |  |  | N/S |  |
|  | **GT** | 60 |  | 144 |  | 154 |  |  | 153 |  | 165 |  |  | 150 |  | 166 |  |  |  |  |
|  | **TT** | 11 |  | 29 |  | 27 |  | 0.77 | 39 |  | 32 |  | 0.54 | 42 |  | 26 |  | 0.07 |  |  |
|  | **Allele** |  |  |  |  |  |  |  |  |  |  |  |  |  |  |  |  |  |  |  |
|  | **T** | 144 | 0.64 | 562 | 0.74 | 558 | 0.73 |  | 501 | 0.68 | 501 | 0.69 |  | 454 | 0.66 | 512 | 0.70 |  |  |  |
|  | **G** | 82 | 0.36 | 202 | 0.26 | 208 | 0.27 | 0.75 | 231 | 0.32 | 229 | 0.31 | 0.94 | 234 | 0.34 | 218 | 0.30 | 0.094 |  |  |
|  | **Call rate (%)** |  |  | 99.5 |  | 99.7 |  |  | 99.7 |  | 99.7 |  |  | 99.4 |  | 99.5 |  |  |  |  |
|  | **HWE (P value)** |  |  | 0.55 |  | 0.75 |  |  | 0.54 |  | 0.34 |  |  | 0.6 |  | 0.1 |  |  |  |  |

|  |  |  |  |  |  |  |  |  |  |  |  |  |  |  |  |  |  |  |  |  |
| --- | --- | --- | --- | --- | --- | --- | --- | --- | --- | --- | --- | --- | --- | --- | --- | --- | --- | --- | --- | --- |
| **Chr 18: 75,282,991** | **rs4799055** |  |  |  |  |  |  |  |  |  |  |  |  |  |  |  |  |  |  |  |
|  | **Genotype** |  |  |  |  |  |  |  |  |  |  |  |  |  |  |  |  |  |  |  |
|  | **TT** | 24 |  | 75 |  | 79 |  |  | 79 |  | 80 |  |  | 90 |  | 88 | 0.31 |  | N/S |  |
|  | **GT** | 57 |  | 205 |  | 201 |  |  | 164 |  | 167 |  |  | 174 |  | 193 | 0.69 |  |  |  |
|  | **GG** | 32 |  | 102 |  | 104 |  | 0.92 | 100 |  | 92 |  | 0.84 | 79 |  | 86 |  | 0.78 |  |  |
|  | **Allele** |  |  |  |  |  |  |  |  |  |  |  |  |  |  |  |  |  |  |  |
|  | **T** | 105 | 0.46 | 355 | 0.46 | 359 | 0.47 |  | 322 | 0.47 | 327 | 0.48 |  | 354 | 0.52 | 369 | 0.50 |  |  |  |
|  | **G** | 121 | 0.54 | 409 | 0.54 | 409 | 0.53 | 0.91 | 364 | 0.53 | 351 | 0.52 | 0.63 | 332 | 0.48 | 365 | 0.50 | 0.62 |  |  |
|  | **Call rate (%)** |  |  | 99.5 |  | 100 |  |  | 99.4 |  | 98.5 |  |  | 99.1 |  | 100 |  |  |  |  |
|  | **HWE (P value)** |  |  | 0.12 |  | 0.31 |  |  | 0.46 |  | 0.8 |  |  | 0.77 |  | 0.32 |  |  |  |  |
|  |  |  |  |  |  |  |  |  |  |  |  |  |  |  |  |  |  |  |  |  |
| **Chr 18: 75,294,649** | **rs2277714** |  |  |  |  |  |  |  |  |  |  |  |  |  |  |  |  |  |  |  |
|  | **Genotype** |  |  |  |  |  |  |  |  |  |  |  |  |  |  |  |  |  |  |  |
|  | **CC** | 111 |  | 382 |  | 384 |  |  | 344 |  | 341 |  |  | 344 |  | 367 |  |  | N/S |  |
|  | **CT** | 1 |  | 0 |  | 0 |  |  | 0 |  | 0 |  |  | 0 |  | 0 |  |  |  |  |
|  | **TT** | 0 |  | 0 |  | 0 |  | N/A | 0 |  | 0 |  | N/A | 0 |  | 0 |  | N/A |  |  |
|  | **Allele** |  |  |  |  |  |  |  |  |  |  |  |  |  |  |  |  |  |  |  |
|  | **C** | 223 | 1.00 | 764 | 1.00 | 768 | 1.00 |  | 688 | 1.00 | 682 | 1.00 |  | 688 | 1.00 | 734 | 1.00 |  |  |  |
|  | **T** | 1 | 0.00 | 0 | 0.00 | 0 | 0.00 | N/A | 0 | 0.00 | 0 | 0.00 | N/A | 0 | 0.00 | 0 | 0.00 | N/A |  |  |
|  | **Call rate (%)** |  |  | 99.5 |  | 100 |  |  | 99.7 |  | 99.1 |  |  | 99.4 |  | 100 |  |  |  |  |
|  | **HWE (P value)** |  |  |  |  |  |  |  | N/A |  | N/A |  |  |  |  |  |  |  |  |  |

|  |  |  |  |  |  |  |  |  |  |  |  |  |  |  |  |  |  |  |  |  |
| --- | --- | --- | --- | --- | --- | --- | --- | --- | --- | --- | --- | --- | --- | --- | --- | --- | --- | --- | --- | --- |
| **Chr 18: 75,312,752** | **rs9966033** |  |  |  |  |  |  |  |  |  |  |  |  |  |  |  |  |  |  |  |
|  | **Genotype** |  |  |  |  |  |  |  |  |  |  |  |  |  |  |  |  |  |  |  |
|  | **TT** | 91 |  | 292 |  | 292 |  |  | 291 |  | 292 |  |  | 251 |  | 271 |  |  | N/S |  |
|  | **CT** | 20 |  | 84 |  | 85 |  |  | 67 |  | 66 |  |  | 89 |  | 87 |  |  |  |  |
|  | **CC** | 2 |  | 6 |  | 7 |  | 0.96 | 5 |  | 6 |  | 0.95 | 4 |  | 7 |  | 0.61 |  |  |
|  | **Allele** |  |  |  |  |  |  |  |  |  |  |  |  |  |  |  |  |  |  |  |
|  | **T** | 202 | 0.89 | 668 | 0.87 | 669 | 0.87 |  | 649 | 0.89 | 650 | 0.89 |  | 591 | 0.86 | 629 | 0.86 |  |  |  |
|  | **C** | 24 | 0.11 | 96 | 0.13 | 99 | 0.13 | 0.85 | 77 | 0.11 | 78 | 0.11 | 0.95 | 97 | 0.14 | 101 | 0.14 | 0.89 |  |  |
|  | **Call rate (%)** |  |  | 99.5 |  | 100 |  |  | 98.9 |  | 99.5 |  |  | 99.4 |  | 99.5 |  |  |  |  |
|  | **HWE (P value)** |  |  | 0.99 |  | 0.78 |  |  | 0.61 |  | 0.32 |  |  | 0.21 |  | 1 |  |  |  |  |
|  |  |  |  |  |  |  |  |  |  |  |  |  |  |  |  |  |  |  |  |  |
| **Chr 18: 75,347,394** | **rs754093** |  |  |  |  |  |  |  |  |  |  |  |  |  |  |  |  |  |  |  |
|  | **Genotype** |  |  |  |  |  |  |  |  |  |  |  |  |  |  |  |  |  |  |  |
|  | **TT** | 34 |  | 94 |  | 111 |  |  | 95 |  | 125 |  |  | 99 |  | 124 |  |  | 0.02 | 0 |
|  | **GT** | 55 |  | 204 |  | 187 |  |  | 184 |  | 154 |  |  | 166 |  | 166 |  |  |  |  |
|  | **GG** | 23 |  | 83 |  | 86 |  | 0.33 | 84 |  | 80 |  | 0.033 | 73 |  | 69 |  | 0.32 |  |  |
|  | **Allele** |  |  |  |  |  |  |  |  |  |  |  |  |  |  |  |  |  |  |  |
|  | **T** | 123 | 0.55 | 392 | 0.51 | 409 | 0.53 |  | 374 | 0.52 | 404 | 0.56 |  | 364 | 0.54 | 414 | 0.58 |  |  |  |
|  | **G** | 101 | 0.45 | 370 | 0.49 | 359 | 0.47 | 0.48 | 352 | 0.48 | 314 | 0.44 | 0.07 | 312 | 0.46 | 304 | 0.42 | 0.15 |  |  |
|  | **Call rate (%)** |  |  | 99.2 |  | 100 |  |  | 98.9 |  | 98.1 |  |  | 97.7 |  | 97.8 |  |  |  |  |
|  | **HWE (P value)** |  |  | 0.16 |  | 0.67 |  |  | 0.78 |  | 0.015 |  |  | 0.83 |  | 0.32 |  |  |  |  |

|  |  |  |  |  |  |  |  |  |  |  |  |  |  |  |  |  |  |  |  |  |
| --- | --- | --- | --- | --- | --- | --- | --- | --- | --- | --- | --- | --- | --- | --- | --- | --- | --- | --- | --- | --- |
| **Chr 18: 75,370,277** | **rs372741** |  |  |  |  |  |  |  |  |  |  |  |  |  |  |  |  |  |  |  |
|  | **Genotype** |  |  |  |  |  |  |  |  |  |  |  |  |  |  |  |  |  |  |  |
|  | **AA** | 56 |  | 188 |  | 187 |  |  | 196 |  | 184 |  |  | 166 |  | 166 |  |  | N/S |  |
|  | **AG** | 45 |  | 165 |  | 160 |  |  | 139 |  | 144 |  |  | 150 |  | 165 |  |  |  |  |
|  | **GG** | 12 |  | 30 |  | 35 |  | 0.79 | 32 |  | 37 |  | 0.66 | 27 |  | 33 |  | 0.71 |  |  |
|  | **Allele** |  |  |  |  |  |  |  |  |  |  |  |  |  |  |  |  |  |  |  |
|  | **A** | 157 | 0.69 | 541 | 0.71 | 534 | 0.70 |  | 531 | 0.72 | 512 | 0.70 |  | 482 | 0.70 | 497 | 0.68 |  |  |  |
|  | **G** | 69 | 0.31 | 225 | 0.29 | 230 | 0.30 | 0.75 | 203 | 0.28 | 218 | 0.30 | 0.35 | 204 | 0.30 | 231 | 0.32 | 0.42 |  |  |
|  | **Call rate (%)** |  |  | 99.7 |  | 99.5 |  |  | 100 |  | 99.7 |  |  | 99.1 |  | 99.2 |  |  |  |  |
|  | **HWE (P value)** |  |  | 0.45 |  | 0.93 |  |  | 0.31 |  | 0.27 |  |  | 0.39 |  | 0.38 |  |  |  |  |
|  |  |  |  |  |  |  |  |  |  |  |  |  |  |  |  |  |  |  |  |  |
| **Chr 18: 75,447,670** | **rs515754** |  |  |  |  |  |  |  |  |  |  |  |  |  |  |  |  |  |  |  |
|  | **Genotype** |  |  |  |  |  |  |  |  |  |  |  |  |  |  |  |  |  |  |  |
|  | **AA** | 36 |  | 89 |  | 99 |  |  | 100 |  | 94 |  |  | 96 |  | 100 |  |  | N/S |  |
|  | **GA** | 59 |  | 192 |  | 194 |  |  | 184 |  | 190 |  |  | 169 |  | 192 |  |  |  |  |
|  | **GG** | 16 |  | 98 |  | 90 |  | 0.65 | 79 |  | 79 |  | 0.87 | 75 |  | 70 |  | 0.6 |  |  |
|  | **Allele** |  |  |  |  |  |  |  |  |  |  |  |  |  |  |  |  |  |  |  |
|  | **A** | 131 | 0.59 | 370 | 0.49 | 392 | 0.51 |  | 384 | 0.53 | 378 | 0.52 |  | 361 | 0.53 | 392 | 0.54 |  |  |  |
|  | **G** | 91 | 0.41 | 388 | 0.51 | 374 | 0.49 | 0.36 | 342 | 0.47 | 348 | 0.48 | 0.75 | 319 | 0.47 | 332 | 0.46 | 0.69 |  |  |
|  | **Call rate (%)** |  |  | 98.7 |  | 99.7 |  |  | 98.9 |  | 99.2 |  |  | 98.3 |  | 98.6 |  |  |  |  |
|  | **HWE (P value)** |  |  | 0.79 |  | 0.79 |  |  | 0.74 |  | 0.35 |  |  | 0.97 |  | 0.2 |  |  |  |  |

|  |  |  |  |  |  |  |  |  |  |  |  |  |  |  |  |  |  |  |  |  |
| --- | --- | --- | --- | --- | --- | --- | --- | --- | --- | --- | --- | --- | --- | --- | --- | --- | --- | --- | --- | --- |
| **Chr 18: 75,456,991** | **rs660749** |  |  |  |  |  |  |  |  |  |  |  |  |  |  |  |  |  |  |  |
|  | **Genotype** |  |  |  |  |  |  |  |  |  |  |  |  |  |  |  |  |  |  |  |
|  | **TT** | 37 |  | 134 |  | 141 |  |  | 136 |  | 120 |  |  | 114 |  | 124 |  |  | N/S |  |
|  | **TC** | 59 |  | 185 |  | 169 |  |  | 158 |  | 170 |  |  | 173 |  | 188 |  |  |  |  |
|  | **CC** | 17 |  | 61 |  | 68 |  | 0.53 | 73 |  | 72 |  | 0.49 | 55 |  | 54 |  | 0.89 |  |  |
|  | **Allele** |  |  |  |  |  |  |  |  |  |  |  |  |  |  |  |  |  |  |  |
|  | **T** | 133 | 0.59 | 453 | 0.60 | 451 | 0.60 |  | 430 | 0.59 | 410 | 0.57 |  | 401 | 0.59 | 436 | 0.60 |  |  |  |
|  | **C** | 93 | 0.41 | 307 | 0.40 | 305 | 0.40 | 0.98 | 304 | 0.41 | 314 | 0.43 | 0.45 | 283 | 0.41 | 296 | 0.40 | 0.72 |  |  |
|  | **Call rate (%)** |  |  | 99 |  | 98.4 |  |  | 100 |  | 98.9 |  |  | 98.8 |  | 99.7 |  |  |  |  |
|  | **HWE (P value)** |  |  | 0.83 |  | 0.17 |  |  | 0.031 |  | 0.4 |  |  | 0.43 |  | 0.2 |  |  |  |  |
|  |  |  |  |  |  |  |  |  |  |  |  |  |  |  |  |  |  |  |  |  |
| **Chr 18: 75,486,867** | **rs7228520** |  |  |  |  |  |  |  |  |  |  |  |  |  |  |  |  |  |  |  |
|  | **Genotype** |  |  |  |  |  |  |  |  |  |  |  |  |  |  |  |  |  |  |  |
|  | **GG** | 48 |  | 154 |  | 164 |  |  | 155 |  | 129 |  |  | 127 |  | 125 |  |  | N/S |  |
|  | **AG** | 52 |  | 179 |  | 163 |  |  | 141 |  | 164 |  |  | 171 |  | 179 |  |  |  |  |
|  | **AA** | 13 |  | 48 |  | 57 |  | 0.4 | 47 |  | 46 |  | 0.13 | 45 |  | 62 |  | 0.34 |  |  |
|  | **Allele** |  |  |  |  |  |  |  |  |  |  |  |  |  |  |  |  |  |  |  |
|  | **G** | 148 | 0.65 | 487 | 0.64 | 491 | 0.64 |  | 451 | 0.66 | 422 | 0.62 |  | 425 | 0.62 | 429 | 0.59 |  |  |  |
|  | **A** | 78 | 0.35 | 275 | 0.36 | 277 | 0.36 | 0.99 | 235 | 0.34 | 256 | 0.38 | 0.18 | 261 | 0.38 | 303 | 0.41 | 0.2 |  |  |
|  | **Call rate (%)** |  |  | 99.2 |  | 100 |  |  | 99.4 |  | 98.5 |  |  | 99.1 |  | 99.7 |  |  |  |  |
|  | **HWE (P value)** |  |  | 0.72 |  | 0.12 |  |  | 0.11 |  | 0.59 |  |  | 0.29 |  | 0.88 |  |  |  |  |

|  |  |  |  |  |  |  |  |  |  |  |  |  |  |  |  |  |  |  |  |  |
| --- | --- | --- | --- | --- | --- | --- | --- | --- | --- | --- | --- | --- | --- | --- | --- | --- | --- | --- | --- | --- |
| **Chr 18: 75,540,520** | **rs3826573** |  |  |  |  |  |  |  |  |  |  |  |  |  |  |  |  |  |  |  |
|  | **Genotype** |  |  |  |  |  |  |  |  |  |  |  |  |  |  |  |  |  |  |  |
|  | **CC** | 38 |  | 132 |  | 125 |  |  | 92 |  | 126 |  |  | 113 |  | 137 |  |  | N/S |  |
|  | **CT** | 47 |  | 174 |  | 180 |  |  | 194 |  | 185 |  |  | 162 |  | 170 |  |  |  |  |
|  | **TT** | 26 |  | 73 |  | 76 |  | 0.84 | 81 |  | 55 |  | 0.0053 | 62 |  | 57 |  | 0.43 |  |  |
|  | **Allele** |  |  |  |  |  |  |  |  |  |  |  |  |  |  |  |  |  |  |  |
|  | **C** | 123 | 0.55 | 438 | 0.58 | 430 | 0.56 |  | 378 | 0.51 | 437 | 0.60 |  | 388 | 0.58 | 444 | 0.61 |  |  |  |
|  | **T** | 99 | 0.45 | 320 | 0.42 | 332 | 0.44 | 0.59 | 356 | 0.49 | 295 | 0.40 | 0.0016 | 286 | 0.42 | 284 | 0.39 | 0.19 |  |  |
|  | **Call rate (%)** |  |  | 98.7 |  | 99.2 |  |  | 100 |  | 100 |  |  | 97.4 |  | 99.2 |  |  |  |  |
|  | **HWE (P value)** |  |  | 0.25 |  | 0.44 |  |  | 0.27 |  | 0.33 |  |  | 0.77 |  | 0.72 |  |  |  |  |
|  |  |  |  |  |  |  |  |  |  |  |  |  |  |  |  |  |  |  |  |  |
| **Chr 18: 75,636,164** | **rs1960120** |  |  |  |  |  |  |  |  |  |  |  |  |  |  |  |  |  |  |  |
|  | **Genotype** |  |  |  |  |  |  |  |  |  |  |  |  |  |  |  |  |  |  |  |
|  | **GG** | 56 |  | 186 |  | 191 |  |  | 186 |  | 155 |  |  | 150 |  | 158 |  |  | N/S |  |
|  | **GA** | 50 |  | 166 |  | 157 |  |  | 133 |  | 148 |  |  | 143 |  | 162 |  |  |  |  |
|  | **AA** | 7 |  | 28 |  | 36 |  | 0.52 | 23 |  | 34 |  | 0.058 | 47 |  | 46 |  | 0.8 |  |  |
|  | **Allele** |  |  |  |  |  |  |  |  |  |  |  |  |  |  |  |  |  |  |  |
|  | **G** | 162 | 0.72 | 538 | 0.71 | 539 | 0.70 |  | 505 | 0.74 | 458 | 0.68 |  | 443 | 0.65 | 478 | 0.65 |  |  |  |
|  | **A** | 64 | 0.28 | 222 | 0.29 | 229 | 0.30 | 0.79 | 179 | 0.26 | 216 | 0.32 | 0.017 | 237 | 0.35 | 254 | 0.35 | 0.95 |  |  |
|  | **Call rate (%)** |  |  | 99 |  | 100 |  |  | 99.1 |  | 98 |  |  | 98.3 |  | 99.7 |  |  |  |  |
|  | **HWE (P value)** |  |  | 0.27 |  | 0.65 |  |  | 0.91 |  | 0.88 |  |  | 0.17 |  | 0.66 |  |  |  |  |

|  |  |  |  |  |  |  |  |  |  |  |  |  |  |  |  |  |  |  |  |  |
| --- | --- | --- | --- | --- | --- | --- | --- | --- | --- | --- | --- | --- | --- | --- | --- | --- | --- | --- | --- | --- |
| **Chr 18: 75,662,966** | **rs8091998** |  |  |  |  |  |  |  |  |  |  |  |  |  |  |  |  |  |  |  |
|  | **Genotype** |  |  |  |  |  |  |  |  |  |  |  |  |  |  |  |  |  |  |  |
|  | **GG** | 69 |  | 234 |  | 218 |  |  | 227 |  | 217 |  |  | 219 |  | 214 |  |  | 0.04 | 0 |
|  | **GT** | 41 |  | 123 |  | 135 |  |  | 104 |  | 115 |  |  | 100 |  | 127 |  |  |  |  |
|  | **TT** | 3 |  | 23 |  | 29 |  | 0.4 | 13 |  | 8 |  | 0.38 | 18 |  | 26 |  | 0.18 |  |  |
|  | **Allele** |  |  |  |  |  |  |  |  |  |  |  |  |  |  |  |  |  |  |  |
|  | **G** | 179 | 0.79 | 591 | 0.78 | 571 | 0.75 |  | 558 | 0.81 | 549 | 0.81 |  | 538 | 0.80 | 555 | 0.76 |  |  |  |
|  | **T** | 47 | 0.21 | 169 | 0.22 | 193 | 0.25 | 0.17 | 130 | 0.19 | 131 | 0.19 | 0.86 | 136 | 0.20 | 179 | 0.24 | 0.058 |  |  |
|  | **Call rate (%)** |  |  | 99 |  | 99.5 |  |  | 99.7 |  | 98.8 |  |  | 97.4 |  | 100 |  |  |  |  |
|  | **HWE (P value)** |  |  | 0.21 |  | 0.21 |  |  | 0.8 |  | 0.11 |  |  | 0.15 |  | 0.24 |  |  |  |  |
|  |  |  |  |  |  |  |  |  |  |  |  |  |  |  |  |  |  |  |  |  |
| **Chr 18: 75,680,761** | **rs7236339** |  |  |  |  |  |  |  |  |  |  |  |  |  |  |  |  |  |  |  |
|  | **Genotype** |  |  |  |  |  |  |  |  |  |  |  |  |  |  |  |  |  |  |  |
|  | **GG** | 70 |  | 239 |  | 209 |  |  | 238 |  | 213 |  |  | 213 |  | 205 |  |  | 0.004 | 0 |
|  | **AG** | 39 |  | 119 |  | 146 |  |  | 89 |  | 117 |  |  | 114 |  | 137 |  |  |  |  |
|  | **AA** | 4 |  | 24 |  | 29 |  | 0.073 | 17 |  | 10 |  | 0.031 | 17 |  | 25 |  | 0.22 |  |  |
|  | **Allele** |  |  |  |  |  |  |  |  |  |  |  |  |  |  |  |  |  |  |  |
|  | **G** | 179 | 0.79 | 597 | 0.78 | 564 | 0.73 |  | 565 | 0.82 | 543 | 0.80 |  | 540 | 0.78 | 547 | 0.75 |  |  |  |
|  | **A** | 47 | 0.21 | 167 | 0.22 | 204 | 0.27 | 0.032 | 123 | 0.18 | 137 | 0.20 | 0.28 | 148 | 0.22 | 187 | 0.25 | 0.078 |  |  |
|  | **Call rate (%)** |  |  | 99.5 |  | 100 |  |  | 99.7 |  | 98.8 |  |  | 99.4 |  | 100 |  |  |  |  |
|  | **HWE (P value)** |  |  | 0.085 |  | 0.62 |  |  | 0.027 |  | 0.2 |  |  | 0.73 |  | 0.75 |  |  |  |  |

|  |  |  |  |  |  |  |  |  |  |  |  |  |  |  |  |  |  |  |  |  |
| --- | --- | --- | --- | --- | --- | --- | --- | --- | --- | --- | --- | --- | --- | --- | --- | --- | --- | --- | --- | --- |
| **Chr 18: 75,693,795** | **rs12960174** |  |  |  |  |  |  |  |  |  |  |  |  |  |  |  |  |  |  |  |
|  | **Genotype** |  |  |  |  |  |  |  |  |  |  |  |  |  |  |  |  |  |  |  |
|  | **CC** | 65 |  | 184 |  | 180 |  |  | 168 |  | 173 |  |  | 173 |  | 174 |  |  | N/S |  |
|  | **TC** | 41 |  | 156 |  | 168 |  |  | 160 |  | 158 |  |  | 131 |  | 154 |  |  |  |  |
|  | **TT** | 6 |  | 41 |  | 35 |  | 0.62 | 39 |  | 33 |  | 0.75 | 39 |  | 39 |  | 0.59 |  |  |
|  | **Allele** |  |  |  |  |  |  |  |  |  |  |  |  |  |  |  |  |  |  |  |
|  | **C** | 171 | 0.76 | 524 | 0.69 | 528 | 0.69 |  | 496 | 0.68 | 504 | 0.69 |  | 477 | 0.70 | 502 | 0.68 |  |  |  |
|  | **T** | 53 | 0.24 | 238 | 0.31 | 238 | 0.31 | 0.95 | 238 | 0.32 | 224 | 0.31 | 0.5 | 209 | 0.30 | 232 | 0.32 | 0.64 |  |  |
|  | **Call rate (%)** |  |  | 99.2 |  | 99.7 |  |  | 100 |  | 99.5 |  |  | 99.1 |  | 100 |  |  |  |  |
|  | **HWE (P value)** |  |  | 0.36 |  | 0.64 |  |  | 0.92 |  | 0.72 |  |  | 0.068 |  | 0.57 |  |  |  |  |
|  |  |  |  |  |  |  |  |  |  |  |  |  |  |  |  |  |  |  |  |  |
| **Chr 18: 75,723,344** | **rs11081569** |  |  |  |  |  |  |  |  |  |  |  |  |  |  |  |  |  |  |  |
|  | **Genotype** |  |  |  |  |  |  |  |  |  |  |  |  |  |  |  |  |  |  |  |
|  | **AA** | 39 |  | 96 |  | 105 |  |  | 84 |  | 97 |  |  | 96 |  | 108 |  |  | N/S |  |
|  | **CA** | 51 |  | 192 |  | 205 |  |  | 185 |  | 181 |  |  | 171 |  | 181 |  |  |  |  |
|  | **CC** | 22 |  | 92 |  | 74 |  | 0.25 | 95 |  | 85 |  | 0.47 | 75 |  | 76 |  | 0.88 |  |  |
|  | **Allele** |  |  |  |  |  |  |  |  |  |  |  |  |  |  |  |  |  |  |  |
|  | **A** | 129 | 0.58 | 384 | 0.51 | 415 | 0.54 |  | 353 | 0.48 | 375 | 0.52 |  | 363 | 0.53 | 397 | 0.54 |  |  |  |
|  | **C** | 95 | 0.42 | 376 | 0.49 | 353 | 0.46 | 0.17 | 375 | 0.52 | 351 | 0.48 | 0.23 | 321 | 0.47 | 333 | 0.46 | 0.62 |  |  |
|  | **Call rate (%)** |  |  | 99 |  | 100 |  |  | 99.2 |  | 99.2 |  |  | 98.8 |  | 99.5 |  |  |  |  |
|  | **HWE (P value)** |  |  | 0.84 |  | 0.14 |  |  | 0.74 |  | 0.97 |  |  | 0.94 |  | 0.99 |  |  |  |  |

|  |  |  |  |  |  |  |  |  |  |  |  |  |  |  |  |  |  |  |  |  |
| --- | --- | --- | --- | --- | --- | --- | --- | --- | --- | --- | --- | --- | --- | --- | --- | --- | --- | --- | --- | --- |
| **Chr 18: 75,776,951** | **rs12964699** |  |  |  |  |  |  |  |  |  |  |  |  |  |  |  |  |  |  |  |
|  | **Genotype** |  |  |  |  |  |  |  |  |  |  |  |  |  |  |  |  |  |  |  |
|  | **CC** | 40 |  | 140 |  | 125 |  |  | 124 |  | 130 |  |  | 126 |  | 142 |  |  | N/S |  |
|  | **GC** | 55 |  | 183 |  | 180 |  |  | 161 |  | 160 |  |  | 157 |  | 160 |  |  |  |  |
|  | **GG** | 17 |  | 57 |  | 79 |  | 0.11 | 59 |  | 51 |  | 0.7 | 60 |  | 63 |  | 0.83 |  |  |
|  | **Allele** |  |  |  |  |  |  |  |  |  |  |  |  |  |  |  |  |  |  |  |
|  | **C** | 135 | 0.60 | 463 | 0.61 | 430 | 0.56 |  | 409 | 0.59 | 420 | 0.62 |  | 409 | 0.60 | 444 | 0.61 |  |  |  |
|  | **G** | 89 | 0.40 | 297 | 0.39 | 338 | 0.44 | 0.051 | 279 | 0.41 | 262 | 0.38 | 0.42 | 277 | 0.40 | 286 | 0.39 | 0.64 |  |  |
|  | **Call rate (%)** |  |  | 99 |  | 100 |  |  | 99.7 |  | 99.1 |  |  | 99.1 |  | 99.5 |  |  |  |  |
|  | **HWE (P value)** |  |  | 0.82 |  | 0.34 |  |  | 0.59 |  | 0.88 |  |  | 0.36 |  | 0.13 |  |  |  |  |
|  |  |  |  |  |  |  |  |  |  |  |  |  |  |  |  |  |  |  |  |  |
| **Chr 18: 75,837,099** | **rs12455687** |  |  |  |  |  |  |  |  |  |  |  |  |  |  |  |  |  |  |  |
|  | **Genotype** |  |  |  |  |  |  |  |  |  |  |  |  |  |  |  |  |  |  |  |
|  | **CC** | 49 |  | 159 |  | 155 |  |  | 153 |  | 146 |  |  | 156 |  | 168 |  |  | N/S |  |
|  | **CT** | 47 |  | 167 |  | 177 |  |  | 139 |  | 148 |  |  | 134 |  | 151 |  |  |  |  |
|  | **TT** | 15 |  | 56 |  | 52 |  | 0.78 | 44 |  | 40 |  | 0.73 | 52 |  | 47 |  | 0.64 |  |  |
|  | **Allele** |  |  |  |  |  |  |  |  |  |  |  |  |  |  |  |  |  |  |  |
|  | **C** | 145 | 0.65 | 485 | 0.63 | 487 | 0.63 |  | 445 | 0.66 | 440 | 0.66 |  | 446 | 0.65 | 487 | 0.67 |  |  |  |
|  | **T** | 77 | 0.35 | 279 | 0.37 | 281 | 0.37 | 0.98 | 227 | 0.34 | 228 | 0.34 | 0.89 | 238 | 0.35 | 245 | 0.33 | 0.6 |  |  |
|  | **Call rate (%)** |  |  | 99.5 |  | 100 |  |  | 97.4 |  | 97.1 |  |  | 98.8 |  | 99.7 |  |  |  |  |
|  | **HWE (P value)** |  |  | 0.26 |  | 0.9 |  |  | 0.17 |  | 0.79 |  |  | 0.012 |  | 0.16 |  |  |  |  |

|  |  |  |  |  |  |  |  |  |  |  |  |  |  |  |  |  |  |  |  |  |
| --- | --- | --- | --- | --- | --- | --- | --- | --- | --- | --- | --- | --- | --- | --- | --- | --- | --- | --- | --- | --- |
| **Chr 18: 75,994,674** | **rs1064059** |  |  |  |  |  |  |  |  |  |  |  |  |  |  |  |  |  |  |  |
|  | **Genotype** |  |  |  |  |  |  |  |  |  |  |  |  |  |  |  |  |  |  |  |
|  | **AA** | 72 |  | 238 |  | 245 |  |  | 209 |  | 214 |  |  | 206 |  | 232 |  |  | N/S |  |
|  | **AG** | 36 |  | 119 |  | 129 |  |  | 111 |  | 111 |  |  | 119 |  | 115 |  |  |  |  |
|  | **GG** | 5 |  | 24 |  | 10 |  | 0.44 | 23 |  | 16 |  | 0.52 | 17 |  | 19 |  | 0.63 |  |  |
|  | **Allele** |  |  |  |  |  |  |  |  |  |  |  |  |  |  |  |  |  |  |  |
|  | **A** | 180 | 0.80 | 595 | 0.78 | 619 | 0.81 |  | 529 | 0.77 | 539 | 0.79 |  | 531 | 0.78 | 579 | 0.79 |  |  |  |
|  | **G** | 46 | 0.20 | 167 | 0.22 | 149 | 0.19 | 0.22 | 157 | 0.23 | 143 | 0.21 | 0.39 | 153 | 0.22 | 153 | 0.21 | 0.5 |  |  |
|  | **Call rate (%)** |  |  | 99.2 |  | 100 |  |  | 99.4 |  | 99.1 |  |  | 98.8 |  | 99.7 |  |  |  |  |
|  | **HWE (P value)** |  |  | 0.088 |  | 0.15 |  |  | 0.12 |  | 0.74 |  |  | 0.97 |  | 0.34 |  |  |  |  |
|  |  |  |  |  |  |  |  |  |  |  |  |  |  |  |  |  |  |  |  |  |
| **Chr 18: 76,043,316** | **rs4799141** |  |  |  |  |  |  |  |  |  |  |  |  |  |  |  |  |  |  |  |
|  | **Genotype** |  |  |  |  |  |  |  |  |  |  |  |  |  |  |  |  |  |  |  |
|  | **CC** | 50 |  | 179 |  | 168 |  |  | 139 |  | 145 |  |  | 156 |  | 166 |  |  | N/S |  |
|  | **TC** | 49 |  | 148 |  | 177 |  |  | 169 |  | 171 |  |  | 142 |  | 155 |  |  |  |  |
|  | **TT** | 15 |  | 53 |  | 38 |  | 0.067 | 56 |  | 48 |  | 0.69 | 43 |  | 44 |  | 0.96 |  |  |
|  | **Allele** |  |  |  |  |  |  |  |  |  |  |  |  |  |  |  |  |  |  |  |
|  | **C** | 149 | 0.66 | 506 | 0.67 | 513 | 0.67 |  | 447 | 0.61 | 461 | 0.63 |  | 454 | 0.67 | 487 | 0.67 |  |  |  |
|  | **T** | 77 | 0.34 | 254 | 0.33 | 253 | 0.33 | 0.87 | 281 | 0.39 | 267 | 0.37 | 0.45 | 228 | 0.33 | 243 | 0.33 | 0.95 |  |  |
|  | **Call rate (%)** |  |  | 99 |  | 99.7 |  |  | 99.2 |  | 99.5 |  |  | 98.6 |  | 99.5 |  |  |  |  |
|  | **HWE (P value)** |  |  | 0.015 |  | 0.38 |  |  | 0.7 |  | 0.83 |  |  | 0.23 |  | 0.4 |  |  |  |  |

|  |  |  |  |  |  |  |  |  |  |  |  |  |  |  |  |  |  |  |  |  |
| --- | --- | --- | --- | --- | --- | --- | --- | --- | --- | --- | --- | --- | --- | --- | --- | --- | --- | --- | --- | --- |
| **Chr 18: 76,069,787** | **rs7243052** |  |  |  |  |  |  |  |  |  |  |  |  |  |  |  |  |  |  |  |
|  | **Genotype** |  |  |  |  |  |  |  |  |  |  |  |  |  |  |  |  |  |  |  |
|  | **GG** | 65 |  | 182 |  | 192 |  |  | 168 |  | 166 |  |  | 175 |  | 192 |  |  | N/S |  |
|  | **AG** | 37 |  | 171 |  | 160 |  |  | 133 |  | 144 |  |  | 130 |  | 151 |  |  |  |  |
|  | **AA** | 11 |  | 28 |  | 32 |  | 0.64 | 43 |  | 31 |  | 0.3 | 36 |  | 23 |  | 0.11 |  |  |
|  | **Allele** |  |  |  |  |  |  |  |  |  |  |  |  |  |  |  |  |  |  |  |
|  | **G** | 167 | 0.74 | 535 | 0.70 | 544 | 0.71 |  | 469 | 0.68 | 476 | 0.70 |  | 480 | 0.70 | 535 | 0.73 |  |  |  |
|  | **A** | 59 | 0.26 | 227 | 0.30 | 224 | 0.29 | 0.79 | 219 | 0.32 | 206 | 0.30 | 0.52 | 202 | 0.30 | 197 | 0.27 | 0.26 |  |  |
|  | **Call rate (%)** |  |  | 99.2 |  | 100 |  |  | 99.7 |  | 99.1 |  |  | 98.6 |  | 99.7 |  |  |  |  |
|  | **HWE (P value)** |  |  | 0.15 |  | 0.87 |  |  | 0.043 |  | 0.98 |  |  | 0.11 |  | 0.35 |  |  |  |  |
|  |  |  |  |  |  |  |  |  |  |  |  |  |  |  |  |  |  |  |  |  |
| **Chr 7: 69,421,134** | **rs12672930** |  |  |  |  |  |  |  |  |  |  |  |  |  |  |  |  |  |  |  |
|  | **Genotype** |  |  |  |  |  |  |  |  |  |  |  |  |  |  |  |  |  |  |  |
|  | **GG** | 22 |  | 115 |  | 150 |  |  | 97 |  | 96 |  |  | 87 |  | 96 |  |  | N/S |  |
|  | **GT** | 53 |  | 193 |  | 165 |  |  | 183 |  | 180 |  |  | 176 |  | 196 |  |  |  |  |
|  | **TT** | 38 |  | 74 |  | 68 |  | 0.029 | 87 |  | 86 |  | 1 | 80 |  | 74 |  | 0.6 |  |  |
|  | **Allele** |  |  |  |  |  |  |  |  |  |  |  |  |  |  |  |  |  |  |  |
|  | **G** | 97 | 0.43 | 423 | 0.55 | 465 | 0.61 |  | 377 | 0.51 | 372 | 0.51 |  | 350 | 0.51 | 388 | 0.53 |  |  |  |
|  | **T** | 129 | 0.57 | 341 | 0.45 | 301 | 0.39 | 0.034 | 357 | 0.49 | 352 | 0.49 | 0.99 | 336 | 0.49 | 344 | 0.47 | 0.45 |  |  |
|  | **Call rate (%)** |  |  | 99.5 |  | 99.7 |  |  | 100 |  | 98.9 |  |  | 99.1 |  | 99.7 |  |  |  |  |
|  | **HWE (P value)** |  |  | 0.66 |  | 0.058 |  |  | 0.97 |  | 0.93 |  |  | 0.62 |  | 0.15 |  |  |  |  |

|  |  |  |  |  |  |  |  |  |  |  |  |  |  |  |  |  |  |  |  |  |
| --- | --- | --- | --- | --- | --- | --- | --- | --- | --- | --- | --- | --- | --- | --- | --- | --- | --- | --- | --- | --- |
| **Chr 7: 69,475,255** | **rs12698902** |  |  |  |  |  |  |  |  |  |  |  |  |  |  |  |  |  |  |  |
|  | **Genotype** |  |  |  |  |  |  |  |  |  |  |  |  |  |  |  |  |  |  |  |
|  | **AA** | 65 |  | 195 |  | 211 |  |  | 183 |  | 207 |  |  | 163 |  | 186 |  |  | N/S |  |
|  | **AG** | 37 |  | 159 |  | 139 |  |  | 141 |  | 131 |  |  | 153 |  | 148 |  |  |  |  |
|  | **GG** | 11 |  | 28 |  | 33 |  | 0.3 | 43 |  | 23 |  | 0.02 | 25 |  | 33 |  | 0.42 |  |  |
|  | **Allele** |  |  |  |  |  |  |  |  |  |  |  |  |  |  |  |  |  |  |  |
|  | **A** | 167 | 0.74 | 549 | 0.72 | 561 | 0.73 |  | 507 | 0.69 | 545 | 0.75 |  | 479 | 0.70 | 520 | 0.71 |  |  |  |
|  | **G** | 59 | 0.26 | 215 | 0.28 | 205 | 0.27 | 0.55 | 227 | 0.31 | 177 | 0.25 | 0.0063 | 203 | 0.30 | 214 | 0.29 | 0.8 |  |  |
|  | **Call rate (%)** |  |  | 99.5 |  | 99.7 |  |  | 100 |  | 98.6 |  |  | 98.6 |  | 100 |  |  |  |  |
|  | **HWE (P value)** |  |  | 0.57 |  | 0.15 |  |  | 0.054 |  | 0.71 |  |  | 0.18 |  | 0.65 |  |  |  |  |
|  |  |  |  |  |  |  |  |  |  |  |  |  |  |  |  |  |  |  |  |  |
| **Chr 7: 69,604,128** | **rs10486872** |  |  |  |  |  |  |  |  |  |  |  |  |  |  |  |  |  |  |  |
|  | **Genotype** |  |  |  |  |  |  |  |  |  |  |  |  |  |  |  |  |  |  |  |
|  | **GG** | 68 |  | 259 |  | 248 |  |  | 227 |  | 227 |  |  | 211 |  | 252 |  |  | N/S |  |
|  | **AG** | 41 |  | 111 |  | 120 |  |  | 122 |  | 108 |  |  | 122 |  | 108 |  |  |  |  |
|  | **AA** | 4 |  | 10 |  | 15 |  | 0.45 | 18 |  | 22 |  | 0.57 | 10 |  | 6 |  | 0.093 |  |  |
|  | **Allele** |  |  |  |  |  |  |  |  |  |  |  |  |  |  |  |  |  |  |  |
|  | **G** | 177 | 0.78 | 629 | 0.83 | 616 | 0.80 |  | 576 | 0.78 | 562 | 0.79 |  | 544 | 0.79 | 612 | 0.84 |  |  |  |
|  | **A** | 49 | 0.22 | 131 | 0.17 | 150 | 0.20 | 0.24 | 158 | 0.22 | 152 | 0.21 | 0.91 | 142 | 0.21 | 120 | 0.16 | 0.037 |  |  |
|  | **Call rate (%)** |  |  | 99 |  | 99.7 |  |  | 100 |  | 97.5 |  |  | 99.1 |  | 99.7 |  |  |  |  |
|  | **HWE (P value)** |  |  | 0.64 |  | 0.92 |  |  | 0.76 |  | 0.066 |  |  | 0.12 |  | 0.14 |  |  |  |  |

|  |  |  |  |  |  |  |  |  |  |  |  |  |  |  |  |  |  |  |  |  |
| --- | --- | --- | --- | --- | --- | --- | --- | --- | --- | --- | --- | --- | --- | --- | --- | --- | --- | --- | --- | --- |
| **Chr 7: 69,648,415** | **rs886661** |  |  |  |  |  |  |  |  |  |  |  |  |  |  |  |  |  |  |  |
|  | **Genotype** |  |  |  |  |  |  |  |  |  |  |  |  |  |  |  |  |  |  |  |
|  | **CC** | 52 |  | 163 |  | 171 |  |  | 158 |  | 162 |  |  | 150 |  | 157 |  |  | N/S |  |
|  | **CT** | 47 |  | 176 |  | 176 |  |  | 161 |  | 162 |  |  | 154 |  | 165 |  |  |  |  |
|  | **TT** | 13 |  | 44 |  | 36 |  | 0.61 | 45 |  | 39 |  | 0.79 | 39 |  | 43 |  | 0.98 |  |  |
|  | **Allele** |  |  |  |  |  |  |  |  |  |  |  |  |  |  |  |  |  |  |  |
|  | **C** | 151 | 0.67 | 502 | 0.66 | 518 | 0.68 |  | 477 | 0.66 | 486 | 0.67 |  | 454 | 0.66 | 479 | 0.66 |  |  |  |
|  | **T** | 73 | 0.33 | 264 | 0.34 | 248 | 0.32 | 0.39 | 251 | 0.34 | 240 | 0.33 | 0.57 | 232 | 0.34 | 251 | 0.34 | 0.82 |  |  |
|  | **Call rate (%)** |  |  | 99.7 |  | 99.7 |  |  | 99.2 |  | 99.2 |  |  | 99.1 |  | 99.5 |  |  |  |  |
|  | **HWE (P value)** |  |  | 0.74 |  | 0.33 |  |  | 0.69 |  | 0.87 |  |  | 0.96 |  | 0.97 |  |  |  |  |
|  |  |  |  |  |  |  |  |  |  |  |  |  |  |  |  |  |  |  |  |  |
| **Chr 7: 69,683,877** | **rs10237317** |  |  |  |  |  |  |  |  |  |  |  |  |  |  |  |  |  |  |  |
|  | **Genotype** |  |  |  |  |  |  |  |  |  |  |  |  |  |  |  |  |  |  |  |
|  | **AA** | 38 |  | 121 |  | 135 |  |  | 131 |  | 128 |  |  | 119 |  | 109 |  |  | N/S |  |
|  | **GA** | 56 |  | 184 |  | 197 |  |  | 165 |  | 173 |  |  | 174 |  | 184 |  |  |  |  |
|  | **GG** | 17 |  | 74 |  | 50 |  | 0.054 | 71 |  | 62 |  | 0.67 | 50 |  | 74 |  | 0.1 |  |  |
|  | **Allele** |  |  |  |  |  |  |  |  |  |  |  |  |  |  |  |  |  |  |  |
|  | **A** | 132 | 0.59 | 426 | 0.56 | 467 | 0.61 |  | 427 | 0.58 | 429 | 0.59 |  | 412 | 0.60 | 402 | 0.55 |  |  |  |
|  | **G** | 90 | 0.41 | 332 | 0.44 | 297 | 0.39 | 0.051 | 307 | 0.42 | 297 | 0.41 | 0.72 | 274 | 0.40 | 332 | 0.45 | 0.044 |  |  |
|  | **Call rate (%)** |  |  | 98.7 |  | 99.5 |  |  | 100 |  | 99.2 |  |  | 99.1 |  | 100 |  |  |  |  |
|  | **HWE (P value)** |  |  | 0.79 |  | 0.096 |  |  | 0.14 |  | 0.79 |  |  | 0.29 |  | 0.82 |  |  |  |  |

|  |  |  |  |  |  |  |  |  |  |  |  |  |  |  |  |  |  |  |  |  |
| --- | --- | --- | --- | --- | --- | --- | --- | --- | --- | --- | --- | --- | --- | --- | --- | --- | --- | --- | --- | --- |
| **Chr 7: 69,691,042** | **rs38319** |  |  |  |  |  |  |  |  |  |  |  |  |  |  |  |  |  |  |  |
|  | **Genotype** |  |  |  |  |  |  |  |  |  |  |  |  |  |  |  |  |  |  |  |
|  | **GG** | 70 |  | 262 |  | 237 |  |  | 242 |  | 235 |  |  | 222 |  | 259 |  |  | N/S |  |
|  | **AG** | 40 |  | 105 |  | 126 |  |  | 111 |  | 113 |  |  | 111 |  | 98 |  |  |  |  |
|  | **AA** | 3 |  | 14 |  | 20 |  | 0.12 | 14 |  | 16 |  | 0.89 | 9 |  | 10 |  | 0.24 |  |  |
|  | **Allele** |  |  |  |  |  |  |  |  |  |  |  |  |  |  |  |  |  |  |  |
|  | **G** | 180 | 0.80 | 629 | 0.83 | 600 | 0.78 |  | 595 | 0.81 | 583 | 0.80 |  | 555 | 0.81 | 616 | 0.84 |  |  |  |
|  | **A** | 46 | 0.20 | 133 | 0.17 | 166 | 0.22 | 0.038 | 139 | 0.19 | 145 | 0.20 | 0.64 | 129 | 0.19 | 118 | 0.16 | 0.17 |  |  |
|  | **Call rate (%)** |  |  | 99.2 |  | 99.7 |  |  | 100 |  | 99.5 |  |  | 98.8 |  | 100 |  |  |  |  |
|  | **HWE (P value)** |  |  | 0.39 |  | 0.54 |  |  | 0.78 |  | 0.61 |  |  | 0.26 |  | 0.84 |  |  |  |  |
|  |  |  |  |  |  |  |  |  |  |  |  |  |  |  |  |  |  |  |  |  |
| **Chr 7: 69,706,550** | **rs38307** |  |  |  |  |  |  |  |  |  |  |  |  |  |  |  |  |  |  |  |
|  | **Genotype** |  |  |  |  |  |  |  |  |  |  |  |  |  |  |  |  |  |  |  |
|  | **AA** | 36 |  | 99 |  | 133 |  |  | 115 |  | 117 |  |  | 111 |  | 97 |  |  | N/S |  |
|  | **AG** | 51 |  | 196 |  | 192 |  |  | 175 |  | 171 |  |  | 170 |  | 186 |  |  |  |  |
|  | **GG** | 26 |  | 88 |  | 58 |  | 0.0037 | 74 |  | 72 |  | 0.97 | 59 |  | 82 |  | 0.1 |  |  |
|  | **Allele** |  |  |  |  |  |  |  |  |  |  |  |  |  |  |  |  |  |  |  |
|  | **A** | 123 | 0.54 | 394 | 0.51 | 458 | 0.60 |  | 405 | 0.56 | 405 | 0.56 |  | 392 | 0.58 | 380 | 0.52 |  |  |  |
|  | **G** | 103 | 0.46 | 372 | 0.49 | 308 | 0.40 | 0.001 | 323 | 0.44 | 315 | 0.44 | 0.81 | 288 | 0.42 | 350 | 0.48 | 0.035 |  |  |
|  | **Call rate (%)** |  |  | 99.7 |  | 99.7 |  |  | 99.2 |  | 98.4 |  |  | 98.3 |  | 99.5 |  |  |  |  |
|  | **HWE (P value)** |  |  | 0.63 |  | 0.4 |  |  | 0.62 |  | 0.51 |  |  | 0.66 |  | 0.69 |  |  |  |  |

|  |  |  |  |  |  |  |  |  |  |  |  |  |  |  |  |  |  |  |  |  |
| --- | --- | --- | --- | --- | --- | --- | --- | --- | --- | --- | --- | --- | --- | --- | --- | --- | --- | --- | --- | --- |
| **Chr 7: 70,518,586** | **rs2066984** |  |  |  |  |  |  |  |  |  |  |  |  |  |  |  |  |  |  |  |
|  | **Genotype** |  |  |  |  |  |  |  |  |  |  |  |  |  |  |  |  |  |  |  |
|  | **AA** | 51 |  | 137 |  | 122 |  |  | 138 |  | 130 |  |  | 132 |  | 139 |  |  | N/S |  |
|  | **AG** | 49 |  | 186 |  | 194 |  |  | 168 |  | 167 |  |  | 155 |  | 172 |  |  |  |  |
|  | **GG** | 13 |  | 58 |  | 67 |  | 0.43 | 61 |  | 67 |  | 0.77 | 56 |  | 55 |  | 0.85 |  |  |
|  | **Allele** |  |  |  |  |  |  |  |  |  |  |  |  |  |  |  |  |  |  |  |
|  | **A** | 151 | 0.67 | 460 | 0.60 | 438 | 0.57 |  | 444 | 0.60 | 427 | 0.59 |  | 419 | 0.61 | 450 | 0.61 |  |  |  |
|  | **G** | 75 | 0.33 | 302 | 0.40 | 328 | 0.43 | 0.21 | 290 | 0.40 | 301 | 0.41 | 0.47 | 267 | 0.39 | 282 | 0.39 | 0.88 |  |  |
|  | **Call rate (%)** |  |  | 99.2 |  | 99.7 |  |  | 100 |  | 99.5 |  |  | 99.1 |  | 99.7 |  |  |  |  |
|  | **HWE (P value)** |  |  | 0.69 |  | 0.5 |  |  | 0.42 |  | 0.3 |  |  | 0.36 |  | 0.88 |  |  |  |  |
|  |  |  |  |  |  |  |  |  |  |  |  |  |  |  |  |  |  |  |  |  |
| **Chr 7: 70,592,167** | **rs7808818** |  |  |  |  |  |  |  |  |  |  |  |  |  |  |  |  |  |  |  |
|  | **Genotype** |  |  |  |  |  |  |  |  |  |  |  |  |  |  |  |  |  |  |  |
|  | **AA** | 43 |  | 109 |  | 103 |  |  | 104 |  | 100 |  |  | 126 |  | 126 |  |  | N/S |  |
|  | **AG** | 56 |  | 204 |  | 194 |  |  | 177 |  | 178 |  |  | 160 |  | 175 |  |  |  |  |
|  | **GG** | 14 |  | 55 |  | 74 |  | 0.2 | 85 |  | 81 |  | 0.95 | 52 |  | 39 |  | N/A |  |  |
|  | **Allele** |  |  |  |  |  |  |  |  |  |  |  |  |  |  |  |  |  |  |  |
|  | **A** | 142 | 0.63 | 422 | 0.57 | 400 | 0.54 |  | 385 | 0.53 | 378 | 0.53 |  | 412 | 0.61 | 427 | 0.63 |  |  |  |
|  | **G** | 84 | 0.37 | 314 | 0.43 | 342 | 0.46 | 0.18 | 347 | 0.47 | 340 | 0.47 | 0.98 | 264 | 0.39 | 253 | 0.37 | N/A |  |  |
|  | **Call rate (%)** |  |  | 95.8 |  | 96.6 |  |  | 99.7 |  | 98.1 |  |  | 97.7 |  | 92.6 |  |  |  |  |
|  | **HWE (P value)** |  |  | 0.011 |  | 0.31 |  |  | 0.56 |  | 0.92 |  |  | 0.92 |  | 0.061 |  |  |  |  |

|  |  |  |  |  |  |  |  |  |  |  |  |  |  |  |  |  |  |  |  |  |
| --- | --- | --- | --- | --- | --- | --- | --- | --- | --- | --- | --- | --- | --- | --- | --- | --- | --- | --- | --- | --- |
| **Chr 7: 70,607,962** | **rs4717599** |  |  |  |  |  |  |  |  |  |  |  |  |  |  |  |  |  |  |  |
|  | **Genotype** |  |  |  |  |  |  |  |  |  |  |  |  |  |  |  |  |  |  |  |
|  | **AA** | 58 |  | 216 |  | 204 |  |  | 194 |  | 198 |  |  | 196 |  | 195 |  |  | N/S |  |
|  | **AG** | 51 |  | 142 |  | 164 |  |  | 154 |  | 139 |  |  | 134 |  | 146 |  |  |  |  |
|  | **GG** | 4 |  | 25 |  | 15 |  | N/A | 19 |  | 26 |  | 0.39 | 13 |  | 26 |  | 0.13 |  |  |
|  | **Allele** |  |  |  |  |  |  |  |  |  |  |  |  |  |  |  |  |  |  |  |
|  | **A** | 167 | 0.74 | 574 | 0.75 | 572 | 0.75 |  | 542 | 0.74 | 535 | 0.74 |  | 526 | 0.77 | 536 | 0.73 |  |  |  |
|  | **G** | 59 | 0.26 | 192 | 0.25 | 194 | 0.25 | N/A | 192 | 0.26 | 191 | 0.26 | 0.95 | 160 | 0.23 | 198 | 0.27 | 0.11 |  |  |
|  | **Call rate (%)** |  |  | 99.7 |  | 99.7 |  |  | 100 |  | 99.2 |  |  | 99.1 |  | 100 |  |  |  |  |
|  | **HWE (P value)** |  |  | 0.8 |  | 0.0097 |  |  | 0.099 |  | 0.81 |  |  | 0.087 |  | 0.85 |  |  |  |  |
|  |  |  |  |  |  |  |  |  |  |  |  |  |  |  |  |  |  |  |  |  |
| **Chr 7: 70,675,868** | **rs11773571** |  |  |  |  |  |  |  |  |  |  |  |  |  |  |  |  |  |  |  |
|  | **Genotype** |  |  |  |  |  |  |  |  |  |  |  |  |  |  |  |  |  |  |  |
|  | **TT** | 52 |  | 175 |  | 163 |  |  | 157 |  | 181 |  |  | 157 |  | 170 |  |  | N/S |  |
|  | **CT** | 50 |  | 169 |  | 170 |  |  | 165 |  | 139 |  |  | 148 |  | 155 |  |  |  |  |
|  | **CC** | 11 |  | 38 |  | 51 |  | 0.31 | 42 |  | 42 |  | 0.14 | 39 |  | 40 |  | 0.97 |  |  |
|  | **Allele** |  |  |  |  |  |  |  |  |  |  |  |  |  |  |  |  |  |  |  |
|  | **T** | 154 | 0.68 | 519 | 0.68 | 496 | 0.65 |  | 479 | 0.66 | 501 | 0.69 |  | 462 | 0.67 | 495 | 0.68 |  |  |  |
|  | **C** | 72 | 0.32 | 245 | 0.32 | 272 | 0.35 | 0.17 | 249 | 0.34 | 223 | 0.31 | 0.17 | 226 | 0.33 | 235 | 0.32 | 0.79 |  |  |
|  | **Call rate (%)** |  |  | 99.5 |  | 100 |  |  | 99.2 |  | 98.9 |  |  | 99.4 |  | 99.5 |  |  |  |  |
|  | **HWE (P value)** |  |  | 0.76 |  | 0.53 |  |  | 0.89 |  | 0.059 |  |  | 0.65 |  | 0.6 |  |  |  |  |

|  |  |  |  |  |  |  |  |  |  |  |  |  |  |  |  |  |  |  |  |  |
| --- | --- | --- | --- | --- | --- | --- | --- | --- | --- | --- | --- | --- | --- | --- | --- | --- | --- | --- | --- | --- |
| **Chr 7: 70,691,674** | **rs2103187** |  |  |  |  |  |  |  |  |  |  |  |  |  |  |  |  |  |  |  |
|  | **Genotype** |  |  |  |  |  |  |  |  |  |  |  |  |  |  |  |  |  |  |  |
|  | **GG** | 47 |  | 130 |  | 142 |  |  | 133 |  | 135 |  |  | 128 |  | 117 |  |  | N/S |  |
|  | **GA** | 54 |  | 190 |  | 168 |  |  | 165 |  | 172 |  |  | 153 |  | 196 |  |  |  |  |
|  | **AA** | 12 |  | 59 |  | 73 |  | 0.19 | 69 |  | 55 |  | 0.43 | 61 |  | 52 |  | 0.056 |  |  |
|  | **Allele** |  |  |  |  |  |  |  |  |  |  |  |  |  |  |  |  |  |  |  |
|  | **G** | 148 | 0.65 | 450 | 0.59 | 452 | 0.59 |  | 431 | 0.59 | 442 | 0.61 |  | 409 | 0.60 | 430 | 0.59 |  |  |  |
|  | **A** | 78 | 0.35 | 308 | 0.41 | 314 | 0.41 | 0.89 | 303 | 0.41 | 282 | 0.39 | 0.36 | 275 | 0.40 | 300 | 0.41 | 0.73 |  |  |
|  | **Call rate (%)** |  |  | 98.7 |  | 99.7 |  |  | 100 |  | 98.9 |  |  | 98.8 |  | 99.5 |  |  |  |  |
|  | **HWE (P value)** |  |  | 0.45 |  | 0.068 |  |  | 0.16 |  | 0.99 |  |  | 0.2 |  | 0.037 |  |  |  |  |
|  |  |  |  |  |  |  |  |  |  |  |  |  |  |  |  |  |  |  |  |  |
| **Chr 7: 70,693,047** | **rs9638627** |  |  |  |  |  |  |  |  |  |  |  |  |  |  |  |  |  |  |  |
|  | **Genotype** |  |  |  |  |  |  |  |  |  |  |  |  |  |  |  |  |  |  |  |
|  | **AA** | 49 |  | 186 |  | 183 |  |  | 176 |  | 179 |  |  | 163 |  | 172 |  |  | N/S |  |
|  | **GA** | 54 |  | 160 |  | 155 |  |  | 163 |  | 145 |  |  | 138 |  | 163 |  |  |  |  |
|  | **GG** | 10 |  | 34 |  | 45 |  | 0.44 | 28 |  | 34 |  | 0.46 | 40 |  | 29 |  | 0.19 |  |  |
|  | **Allele** |  |  |  |  |  |  |  |  |  |  |  |  |  |  |  |  |  |  |  |
|  | **A** | 152 | 0.67 | 532 | 0.70 | 521 | 0.68 |  | 515 | 0.70 | 503 | 0.70 |  | 464 | 0.68 | 507 | 0.70 |  |  |  |
|  | **G** | 74 | 0.33 | 228 | 0.30 | 245 | 0.32 | 0.4 | 219 | 0.30 | 213 | 0.30 | 0.97 | 218 | 0.32 | 221 | 0.30 | 0.51 |  |  |
|  | **Call rate (%)** |  |  | 99 |  | 99.7 |  |  | 100 |  | 97.8 |  |  | 98.6 |  | 99.2 |  |  |  |  |
|  | **HWE (P value)** |  |  | 0.96 |  | 0.17 |  |  | 0.24 |  | 0.56 |  |  | 0.2 |  | 0.26 |  |  |  |  |

|  |  |  |  |  |  |  |  |  |  |  |  |  |  |  |  |  |  |  |  |  |
| --- | --- | --- | --- | --- | --- | --- | --- | --- | --- | --- | --- | --- | --- | --- | --- | --- | --- | --- | --- | --- |
| **Chr 7: 70,709,896** | **rs9638296** |  |  |  |  |  |  |  |  |  |  |  |  |  |  |  |  |  |  |  |
|  | **Genotype** |  |  |  |  |  |  |  |  |  |  |  |  |  |  |  |  |  |  |  |
|  | **CC** | 30 |  | 91 |  | 100 |  |  | 67 |  | 86 |  |  | 84 |  | 100 |  |  | N/S |  |
|  | **GC** | 57 |  | 186 |  | 187 |  |  | 196 |  | 179 |  |  | 186 |  | 175 |  |  |  |  |
|  | **GG** | 25 |  | 104 |  | 97 |  | 0.72 | 101 |  | 99 |  | 0.21 | 74 |  | 85 |  | 0.35 |  |  |
|  | **Allele** |  |  |  |  |  |  |  |  |  |  |  |  |  |  |  |  |  |  |  |
|  | **C** | 117 | 0.52 | 368 | 0.48 | 387 | 0.50 |  | 330 | 0.45 | 351 | 0.48 |  | 354 | 0.51 | 375 | 0.52 |  |  |  |
|  | **G** | 107 | 0.48 | 394 | 0.52 | 381 | 0.50 | 0.41 | 398 | 0.55 | 377 | 0.52 | 0.27 | 334 | 0.49 | 345 | 0.48 | 0.81 |  |  |
|  | **Call rate (%)** |  |  | 99.2 |  | 100 |  |  | 99.2 |  | 99.5 |  |  | 99.4 |  | 98.1 |  |  |  |  |
|  | **HWE (P value)** |  |  | 0.66 |  | 0.61 |  |  | 0.099 |  | 0.77 |  |  | 0.13 |  | 0.62 |  |  |  |  |
|  |  |  |  |  |  |  |  |  |  |  |  |  |  |  |  |  |  |  |  |  |
| **Chr 7: 70,718,338** | **rs10807735** |  |  |  |  |  |  |  |  |  |  |  |  |  |  |  |  |  |  |  |
|  | **Genotype** |  |  |  |  |  |  |  |  |  |  |  |  |  |  |  |  |  |  |  |
|  | **AA** | 30 |  | 91 |  | 99 |  |  | 67 |  | 88 |  |  | 82 |  | 106 |  |  | N/S |  |
|  | **AG** | 56 |  | 183 |  | 188 |  |  | 200 |  | 178 |  |  | 184 |  | 174 |  |  |  |  |
|  | **GG** | 26 |  | 105 |  | 97 |  | 0.71 | 96 |  | 99 |  | 0.12 | 74 |  | 82 |  | 0.22 |  |  |
|  | **Allele** |  |  |  |  |  |  |  |  |  |  |  |  |  |  |  |  |  |  |  |
|  | **A** | 116 | 0.52 | 365 | 0.48 | 386 | 0.50 |  | 334 | 0.46 | 354 | 0.48 |  | 348 | 0.51 | 386 | 0.53 |  |  |  |
|  | **G** | 108 | 0.48 | 393 | 0.52 | 382 | 0.50 | 0.41 | 392 | 0.54 | 376 | 0.52 | 0.34 | 332 | 0.49 | 338 | 0.47 | 0.42 |  |  |
|  | **Call rate (%)** |  |  | 98.7 |  | 100 |  |  | 98.9 |  | 99.7 |  |  | 98.3 |  | 98.6 |  |  |  |  |
|  | **HWE (P value)** |  |  | 0.52 |  | 0.68 |  |  | 0.038 |  | 0.65 |  |  | 0.13 |  | 0.51 |  |  |  |  |

|  |  |  |  |  |  |  |  |  |  |  |  |  |  |  |  |  |  |  |  |  |
| --- | --- | --- | --- | --- | --- | --- | --- | --- | --- | --- | --- | --- | --- | --- | --- | --- | --- | --- | --- | --- |
| **Chr 7: 70,726,671** | **rs3857700** |  |  |  |  |  |  |  |  |  |  |  |  |  |  |  |  |  |  |  |
|  | **Genotype** |  |  |  |  |  |  |  |  |  |  |  |  |  |  |  |  |  |  |  |
|  | **AA** | 39 |  | 160 |  | 152 |  |  | 158 |  | 155 |  |  | 136 |  | 152 |  |  | N/S |  |
|  | **AG** | 61 |  | 175 |  | 175 |  |  | 173 |  | 164 |  |  | 163 |  | 171 |  |  |  |  |
|  | **GG** | 13 |  | 46 |  | 57 |  | 0.5 | 33 |  | 45 |  | 0.35 | 41 |  | 41 |  | 0.88 |  |  |
|  | **Allele** |  |  |  |  |  |  |  |  |  |  |  |  |  |  |  |  |  |  |  |
|  | **A** | 139 | 0.62 | 495 | 0.65 | 479 | 0.62 |  | 489 | 0.67 | 474 | 0.65 |  | 435 | 0.64 | 475 | 0.65 |  |  |  |
|  | **G** | 87 | 0.38 | 267 | 0.35 | 289 | 0.38 | 0.29 | 239 | 0.33 | 254 | 0.35 | 0.41 | 245 | 0.36 | 253 | 0.35 | 0.62 |  |  |
|  | **Call rate (%)** |  |  | 99.2 |  | 100 |  |  | 99.2 |  | 99.5 |  |  | 98.3 |  | 99.2 |  |  |  |  |
|  | **HWE (P value)** |  |  | 0.86 |  | 0.57 |  |  | 0.14 |  | 0.87 |  |  | 0.46 |  | 0.49 |  |  |  |  |
|  |  |  |  |  |  |  |  |  |  |  |  |  |  |  |  |  |  |  |  |  |
| **Chr 7: 70,745,160** | **rs4719155** |  |  |  |  |  |  |  |  |  |  |  |  |  |  |  |  |  |  |  |
|  | **Genotype** |  |  |  |  |  |  |  |  |  |  |  |  |  |  |  |  |  |  |  |
|  | **GG** | 52 |  | 191 |  | 184 |  |  | 178 |  | 192 |  |  | 162 |  | 181 |  |  | N/S |  |
|  | **AG** | 53 |  | 157 |  | 156 |  |  | 157 |  | 140 |  |  | 154 |  | 151 |  |  |  |  |
|  | **AA** | 8 |  | 34 |  | 44 |  | 0.49 | 29 |  | 30 |  | 0.47 | 27 |  | 30 |  | 0.69 |  |  |
|  | **Allele** |  |  |  |  |  |  |  |  |  |  |  |  |  |  |  |  |  |  |  |
|  | **G** | 157 | 0.69 | 539 | 0.71 | 524 | 0.68 |  | 513 | 0.70 | 524 | 0.72 |  | 478 | 0.70 | 513 | 0.71 |  |  |  |
|  | **A** | 69 | 0.31 | 225 | 0.29 | 244 | 0.32 | 0.32 | 215 | 0.30 | 200 | 0.28 | 0.42 | 208 | 0.30 | 211 | 0.29 | 0.63 |  |  |
|  | **Call rate (%)** |  |  | 99.5 |  | 100 |  |  | 99.2 |  | 98.9 |  |  | 99.1 |  | 98.6 |  |  |  |  |
|  | **HWE (P value)** |  |  | 0.83 |  | 0.22 |  |  | 0.49 |  | 0.53 |  |  | 0.25 |  | 0.85 |  |  |  |  |

|  |  |  |  |  |  |  |  |  |  |  |  |  |  |  |  |  |  |  |  |  |
| --- | --- | --- | --- | --- | --- | --- | --- | --- | --- | --- | --- | --- | --- | --- | --- | --- | --- | --- | --- | --- |
| **Chr 7: 70,763,184** | **rs605586** |  |  |  |  |  |  |  |  |  |  |  |  |  |  |  |  |  |  |  |
|  | **Genotype** |  |  |  |  |  |  |  |  |  |  |  |  |  |  |  |  |  |  |  |
|  | **CC** | 41 |  | 149 |  | 141 |  |  | 143 |  | 154 |  |  | 122 |  | 149 |  |  | N/S |  |
|  | **TC** | 53 |  | 174 |  | 180 |  |  | 173 |  | 154 |  |  | 167 |  | 163 |  |  |  |  |
|  | **TT** | 18 |  | 59 |  | 62 |  | 0.82 | 51 |  | 56 |  | 0.42 | 54 |  | 54 |  | 0.37 |  |  |
|  | **Allele** |  |  |  |  |  |  |  |  |  |  |  |  |  |  |  |  |  |  |  |
|  | **C** | 135 | 0.60 | 472 | 0.62 | 462 | 0.60 |  | 459 | 0.63 | 462 | 0.63 |  | 411 | 0.60 | 461 | 0.63 |  |  |  |
|  | **T** | 89 | 0.40 | 292 | 0.38 | 304 | 0.40 | 0.56 | 275 | 0.37 | 266 | 0.37 | 0.71 | 275 | 0.40 | 271 | 0.37 | 0.24 |  |  |
|  | **Call rate (%)** |  |  | 99.5 |  | 99.7 |  |  | 100 |  | 99.5 |  |  | 99.1 |  | 99.7 |  |  |  |  |
|  | **HWE (P value)** |  |  | 0.49 |  | 0.72 |  |  | 0.91 |  | 0.094 |  |  | 0.8 |  | 0.39 |  |  |  |  |
|  |  |  |  |  |  |  |  |  |  |  |  |  |  |  |  |  |  |  |  |  |
| **Chr 7: 70,773,128** | **rs2302440** |  |  |  |  |  |  |  |  |  |  |  |  |  |  |  |  |  |  |  |
|  | **Genotype** |  |  |  |  |  |  |  |  |  |  |  |  |  |  |  |  |  |  |  |
|  | **GG** | 33 |  | 120 |  | 118 |  |  | 106 |  | 104 |  |  | 95 |  | 106 |  |  | N/S |  |
|  | **AG** | 52 |  | 182 |  | 179 |  |  | 186 |  | 181 |  |  | 177 |  | 193 |  |  |  |  |
|  | **AA** | 28 |  | 79 |  | 86 |  | 0.85 | 75 |  | 79 |  | 0.91 | 70 |  | 68 |  | 0.8 |  |  |
|  | **Allele** |  |  |  |  |  |  |  |  |  |  |  |  |  |  |  |  |  |  |  |
|  | **G** | 118 | 0.52 | 422 | 0.55 | 415 | 0.54 |  | 398 | 0.54 | 389 | 0.53 |  | 367 | 0.54 | 405 | 0.55 |  |  |  |
|  | **A** | 108 | 0.48 | 340 | 0.45 | 351 | 0.46 | 0.64 | 336 | 0.46 | 339 | 0.47 | 0.76 | 317 | 0.46 | 329 | 0.45 | 0.57 |  |  |
|  | **Call rate (%)** |  |  | 99.2 |  | 99.7 |  |  | 100 |  | 99.5 |  |  | 98.8 |  | 100 |  |  |  |  |
|  | **HWE (P value)** |  |  | 0.51 |  | 0.25 |  |  | 0.69 |  | 0.99 |  |  | 0.45 |  | 0.23 |  |  |  |  |

|  |  |  |  |  |  |  |  |  |  |  |  |  |  |  |  |  |  |  |  |  |
| --- | --- | --- | --- | --- | --- | --- | --- | --- | --- | --- | --- | --- | --- | --- | --- | --- | --- | --- | --- | --- |
| **Chr 7: 70,788,043** | **rs845056** |  |  |  |  |  |  |  |  |  |  |  |  |  |  |  |  |  |  |  |
|  | **Genotype** |  |  |  |  |  |  |  |  |  |  |  |  |  |  |  |  |  |  |  |
|  | **GG** | 50 |  | 155 |  | 154 |  |  | 155 |  | 157 |  |  | 137 |  | 143 |  |  | N/S |  |
|  | **AG** | 49 |  | 171 |  | 167 |  |  | 174 |  | 153 |  |  | 157 |  | 169 |  |  |  |  |
|  | **AA** | 14 |  | 56 |  | 62 |  | 0.84 | 38 |  | 55 |  | 0.11 | 49 |  | 53 |  | 0.98 |  |  |
|  | **Allele** |  |  |  |  |  |  |  |  |  |  |  |  |  |  |  |  |  |  |  |
|  | **G** | 149 | 0.66 | 481 | 0.63 | 475 | 0.62 |  | 484 | 0.66 | 467 | 0.64 |  | 431 | 0.63 | 455 | 0.62 |  |  |  |
|  | **A** | 77 | 0.34 | 283 | 0.37 | 291 | 0.38 | 0.7 | 250 | 0.34 | 263 | 0.36 | 0.43 | 255 | 0.37 | 275 | 0.38 | 0.85 |  |  |
|  | **Call rate (%)** |  |  | 99.5 |  | 99.7 |  |  | 100 |  | 99.7 |  |  | 99.1 |  | 99.5 |  |  |  |  |
|  | **HWE (P value)** |  |  | 0.43 |  | 0.14 |  |  | 0.29 |  | 0.083 |  |  | 0.71 |  | 0.79 |  |  |  |  |
|  |  |  |  |  |  |  |  |  |  |  |  |  |  |  |  |  |  |  |  |  |
| **Chr 7: 70,823,173** | **rs620269** |  |  |  |  |  |  |  |  |  |  |  |  |  |  |  |  |  |  |  |
|  | **Genotype** |  |  |  |  |  |  |  |  |  |  |  |  |  |  |  |  |  |  |  |
|  | **GG** | 44 |  | 154 |  | 128 |  |  | 128 |  | 135 |  |  | 119 |  | 131 |  |  | N/S |  |
|  | **GA** | 43 |  | 171 |  | 202 |  |  | 175 |  | 177 |  |  | 159 |  | 169 |  |  |  |  |
|  | **AA** | 26 |  | 57 |  | 53 |  | 0.077 | 64 |  | 52 |  | 0.49 | 65 |  | 67 |  | 0.95 |  |  |
|  | **Allele** |  |  |  |  |  |  |  |  |  |  |  |  |  |  |  |  |  |  |  |
|  | **G** | 131 | 0.58 | 479 | 0.63 | 458 | 0.60 |  | 431 | 0.59 | 447 | 0.61 |  | 397 | 0.58 | 431 | 0.59 |  |  |  |
|  | **A** | 95 | 0.42 | 285 | 0.37 | 308 | 0.40 | 0.24 | 303 | 0.41 | 281 | 0.39 | 0.3 | 289 | 0.42 | 303 | 0.41 | 0.75 |  |  |
|  | **Call rate (%)** |  |  | 99.5 |  | 99.7 |  |  | 100 |  | 99.5 |  |  | 99.1 |  | 100 |  |  |  |  |
|  | **HWE (P value)** |  |  | 0.4 |  | 0.058 |  |  | 0.75 |  | 0.62 |  |  | 0.36 |  | 0.34 |  |  |  |  |

|  |  |  |  |  |  |  |  |  |  |  |  |  |  |  |  |  |  |  |  |  |
| --- | --- | --- | --- | --- | --- | --- | --- | --- | --- | --- | --- | --- | --- | --- | --- | --- | --- | --- | --- | --- |
| **Chr 7: 70,870,061** | **rs4719176** |  |  |  |  |  |  |  |  |  |  |  |  |  |  |  |  |  |  |  |
|  | **Genotype** |  |  |  |  |  |  |  |  |  |  |  |  |  |  |  |  |  |  |  |
|  | **CC** | 54 |  | 182 |  | 177 |  |  | 170 |  | 171 |  |  | 165 |  | 162 |  |  | N/S |  |
|  | **TC** | 43 |  | 156 |  | 162 |  |  | 166 |  | 161 |  |  | 137 |  | 159 |  |  |  |  |
|  | **TT** | 14 |  | 45 |  | 45 |  | 0.91 | 27 |  | 28 |  | 0.96 | 40 |  | 42 |  | 0.58 |  |  |
|  | **Allele** |  |  |  |  |  |  |  |  |  |  |  |  |  |  |  |  |  |  |  |
|  | **C** | 151 | 0.68 | 520 | 0.68 | 516 | 0.67 |  | 506 | 0.70 | 503 | 0.70 |  | 467 | 0.68 | 483 | 0.67 |  |  |  |
|  | **T** | 71 | 0.32 | 246 | 0.32 | 252 | 0.33 | 0.77 | 220 | 0.30 | 217 | 0.30 | 0.95 | 217 | 0.32 | 243 | 0.33 | 0.48 |  |  |
|  | **Call rate (%)** |  |  | 99.7 |  | 100 |  |  | 98.9 |  | 98.4 |  |  | 98.8 |  | 98.9 |  |  |  |  |
|  | **HWE (P value)** |  |  | 0.2 |  | 0.4 |  |  | 0.12 |  | 0.24 |  |  | 0.16 |  | 0.75 |  |  |  |  |

Footnote: Genotype and allele counts for the three European cohorts analysed in the replication study are presented alongside Hapmap CEU reference genotype and allele counts which refer to those from the CEPH collection and represent data collected from Utah residents with Northern and Western European ancestry. Allele frequencies (freq) are also presented. Significant results (P value <0.05) are highlighted in yellow. Results excluded on the basis of a genotyping call rate of <95% are highlighted in red. Results excluded on the basis of the control cohort being out of HWE (P<0.05) are highlighted in blue. P values are not reported for excluded SNPs (marked N/A). The Swedish cohort comprised 367 AAD cases and 366 controls except where highlighted in peach where the cohort comprised 345 AAD cases and 344 controls due to DNA shortage. N/S meta-analysis results not reaching statistical significance (P value >0.05). The I^2^ statistic gives an estimate of heterogeneity. An I^2^ of 0% indicates no genetic heterogeneity between the tested cohorts.
